# Autocorrelation in category judgement

**DOI:** 10.1177/17470218231159393

**Published:** 2023-03-24

**Authors:** Donald Laming

**Affiliations:** Department of Psychology, University of Cambridge, Cambridge, UK

**Keywords:** Autocorrelation, category judgement, information transmitted, magnitude estimation, relativity of judgement

## Abstract

Jesteadt et al. discovered a remarkable pattern of autocorrelation in log estimates of loudness. Responses to repeated stimuli correlated to about +0.7, but that correlation was much reduced (0.1) following large differences between successive stimuli. The experiment reported here demonstrates the same pattern in absolute identification without feedback; if feedback is supplied, the pattern is much muted. A model is proposed for this pattern of autocorrelation, based on the premise: “There is no absolute judgment of sensory magnitudes; nor is there any absolute judgment of differences/ratios between sensory magnitudes.” Each stimulus in an experiment is compared with its predecessor, greater, less than, or about the same. The *variability* of that comparison increases with the difference in magnitude between the stimuli, so the assessment of a stimulus far removed from its predecessor is very uncertain. The model provides explanations for the apparent normal variability of sensory stimuli, for the “bow” effect and for the widely reported pattern of sequential effects. It has applications to the effects of stimulus range, to the difficulty of identifying more than five stimuli on a single continuum without error, and to inspection tasks in general, notably medical screening and the marking of examination scripts.

In the course of a routine regression analysis of log magnitude estimates of 1 kHz tones on previous stimuli and responses, Jesteadt et al. (1977, Figure 4) discovered a remarkable pattern of autocorrelation. If a stimulus was repeated (to within 4 dB) on successive trials, successive log estimates correlated about +0.7, but that correlation was much reduced (0.1) following large differences between successive stimuli (see Figure 3). This pattern has been replicated by Green et al. (1977), Luce and Green (1978), Baird et al. (1980), and Ward (1979), but only in magnitude estimation, production, and matching tasks. The experiment reported here tests whether a similar pattern of autocorrelation obtains in category judgement. It bears on two separate questions:

1. Braida and Durlach (1972, Expt. 1) compared magnitude estimation (ME) with absolute identification (AI without feedback), using the same set of stimuli and the same schedule of presentation for both tasks. Their published analysis suggests strongly that ME and AI are essentially the same task, differing only in the mode of expressing the judgements. If that is so, then AI should show the same pattern of autocorrelation as ME; more importantly, if AI *fails* to show that pattern, then ME and AI are *different* tasks. The experiment reported here replicates Braida and Durlach’s Experiment 1, with an extra condition, AI with immediate knowledge of results (FB).
2. Stewart et al. (2005) published a relative judgement model of AI in which judgements are based on the difference (or ratio) between the present and the preceding stimulus. This has proved controversial (e.g., S. D. Brown et al., 2009; S. D. Brown, Marley, & Lacouture, 2007), because the discrimination between two fixed stimuli (e.g., two 0.5-s 1 kHz tones at 60 and 62 dB, Lockhead & Hinson, 1986) is impaired by the inclusion of a more remote stimulus (54 dB) in the identification task. This would appear to involve some absolute assessment of the difference between 54 and 60 dB. In addition, Guest et al. (2016) questioned the role of relative judgement on the basis of a comparison between two tasks: one a conventional AI (of line length) and the second a similar AI of the *difference* between successive lengths of line (see also Guest et al., 2018).

Anticipating the results of this present experiment, AI (without feedback) shows the same pattern of autocorrelation as ME. Psychologically, they are the same task, differing only in details. Second, the debate (2. above), proposing relative judgement instead of AI, is predicated on an *absolute* judgement of the *difference* between successive stimuli. The results below point to a *third* idea, not previously considered (except Laming, 1984, 1997). Each stimulus in an experiment is compared with its predecessor, greater, less than, or about the same. The *variability* of that comparison increases with the difference in magnitude between the stimuli, so the assessment of a stimulus far removed from its predecessor is very uncertain. This idea accommodates the pattern of autocorrelation (1. above), which, in turn, proves incompatible with an *absolute* judgement of the difference between successive stimuli.

Previous authors have proposed a variety of structures in memory to support an AI of the stimulus and those structures have introduced a number of unnecessary problems in the modelling of the “bow” effect (*d*′ between adjacent stimuli is depressed in the centre of the stimulus range; Luce et al., 1982; Weber et al., 1977) and the widely reported pattern of sequential effects in category judgement (e.g., Ward & Lockhead, 1970, 1971; see Stewart et al., 2005, esp. Table 2, p. 886, for a review). The analyses below relate the bow effect and the sequential effects directly to the pattern of autocorrelation. Structures in memory to support an absolute comparison are not needed.

## The experiment

### Stimuli

The stimuli were 10, 500-ms bursts of 1,000 Hz tone at levels ranging from 50 to 86 dB, at 4 dB spacing. They were presented at 7-s intervals. The presentation was programmed on a BBC microcomputer, which actuated a Farnell FG1 function generator set to 1 kHz and a Grason–Stadler digital attenuator according to the stimulus level required. The 500-ms tone burst was then passed through a Bruel & Kjoer bandpass filter set to 1/3 octave at 1 kHz, before being delivered to the observer through a Pioneer amplifier and binaural headphones (Radio Spares, 8 Ω impedance). The experiment took place in a sound-proof chamber.

### Procedure

ME always came first on the ground that observers should not know that there were only 10 different levels of tone, lest it bias their numerical estimates. AI without feedback always came second on the ground that 3,000 trials with feedback might improve otherwise untrained performance (cf. Mori & Ward, 1995; Tanner et al., 1970). There were 3,000 trials under each condition, administered in five 2-hr sessions on successive days of the week, at the same time each day, Monday–Friday. There were 6 blocks of 100 stimuli in each session. The BBC microcomputer delivered a different random sequence of stimuli for each block of 100 trials, subject to the constraint that each stimulus value followed each other stimulus value (including itself) exactly once. The three different conditions were administered in successive weeks.

In the ME condition, the fifth most intense stimulus (70 dB) was presented 10 times (at 7-s intervals) before each block of 100 trials. The observers were instructed to assign the number 100 to the loudness of this stimulus and to report the numerical loudnesses of subsequent stimuli by using a ratio scale: “Stimuli which appear twice as loud should be reported as 200, while stimuli which appear half as loud should be reported as 50.” In the AI and FB conditions the stimuli were presented once (at 7-s intervals) in order of decreasing intensity before each block of 100 trials. The observers were told to identify the most intense stimulus as 10, the second most intense as 9, and so on, and were instructed to assign these numbers to the stimuli presented on subsequent trials as accurately as possible.

Each trial began with a visual warning “GET READY” for 1 s, followed by the stimulus for 0.5 s. The subject was then instructed “Type response number” and the computer waited until a total of 7 s had passed before presenting the next visual warning. In the FB condition the screen showed “Stimulus was number x” for the last 2 s of that 7-s period.

### Instructions

For the ME condition the instructions included:

This experiment is about how people judge the loudness of sounds—not the physical energy in the sound, but the loudness as you experience it. In the earphones you will hear a series of tones, all of them of the same pitch, but of various loudnesses. I want you to put a number to each tone in proportion to how loud it seems to you. The first ten tones will all be of the same loudness and you are to call that loudness 100 units. Thereafter, the computer will give you whatever loudness of tone it thinks fit; except that the tone will never be so loud as to hurt your ears, nor so quiet that you are unable to hear it. If one of those tones sounds three times as loud as the standard loudness, you are to call it 300; and if some other tone sounds only one quarter as loud, call it 25. In case this seems an obscure task to you, here are some slips of paper with a line drawn on each. The first line is 100 units long; write a number against each succeeding line to say how long it seems to you in relation to that first line.^1^ Remember, it is not the physical length of the line that matters, but how long it seems to you.

For the AI condition the instructions included:

There are ten different loudnesses altogether and they will be numbered 1 to 10. I want you to say which loudness you think each tone is. To help you, the first ten tones will be the ten different loudnesses in descending order from 10 to 1, and the correct response to these tones will be 10, 9, 8, etc.^2^ Thereafter, the computer will give you any one of those ten loudnesses selected at random, and you are to type in whatever number in the range 1 to 10 you think corresponds to that particular loudness.

For the FB condition the instructions added:

When you have typed in your number, the computer will tell you what the correct answer was.

This replication enabled study of all the principal phenomena of AI and ME within the one experiment, thereby excluding the possible involvement of differences in stimuli, instrumentation, and personnel.

### Observers

There were four observers, two university students, and two 18-year-old school-leavers, about to go to university. All participated on a voluntary basis, and the experiment was conducted in accordance with the ethical standards of the 1975 Helsinki Declaration. They were each paid £60 on completion of the experiment.^3^

### Terminology

The experiment presents stimuli from a set of 10, designated *S_i_, i* = 1, . . . ., 10, in ascending order of magnitude. The stimulus mean on the scale of 1–10 (5.5) will be designated by $\bar{S}$ ; the variance about that mean is 8.25. Subscripts *i* and *j* will denote particular stimuli from this set, while subscript *n* (*n* + 1, etc.) will denote the stimulus presented on the *n*th trial. In ME *N_n_*, usually log *N_n_*, will denote the numerical assignment on the *n*th trial; in AI and FB, the observed response on the *n*th trial will be designated by ***R***_*n*_ (bold face). The analysis below typically generates predictions, *R_n_* (italic) for trial *n*, where *R_n_* is not constrained to match any of the stimuli. Such predictions have to be evaluated by comparison with the recorded data. In principle, log *N_n_* may take any positive value, so there is not obviously a problem with ME; but *R_n_* has to correspond somehow to one of the stimuli. This presents a potential source of confusion, resolved in this manner: The predicted response on the *n*th trial (any value) will be designated by *R_n_* (italic), while the response actually observed (necessarily 1, . . . 10) by ***R***_*n*_ (bold face).

Equation 1 (below) introduces *B_n_*, a random variable, and ε_*n*_, an error term of zero mean. The suffix *n* signifies that the values of these variables are specific to trial *n*, while their distributions are common to all trials. The mean of *B_n_* will be denoted by β, its variance by σ_β_^2^, and the variance of ε_*n*_ by σ_ε_^2^. Equation 4 introduces a “prior expectation,” which carries weight (1-θ).

## Results^4^

### Comparison of ME, AI, and FB

**Figure 1.**
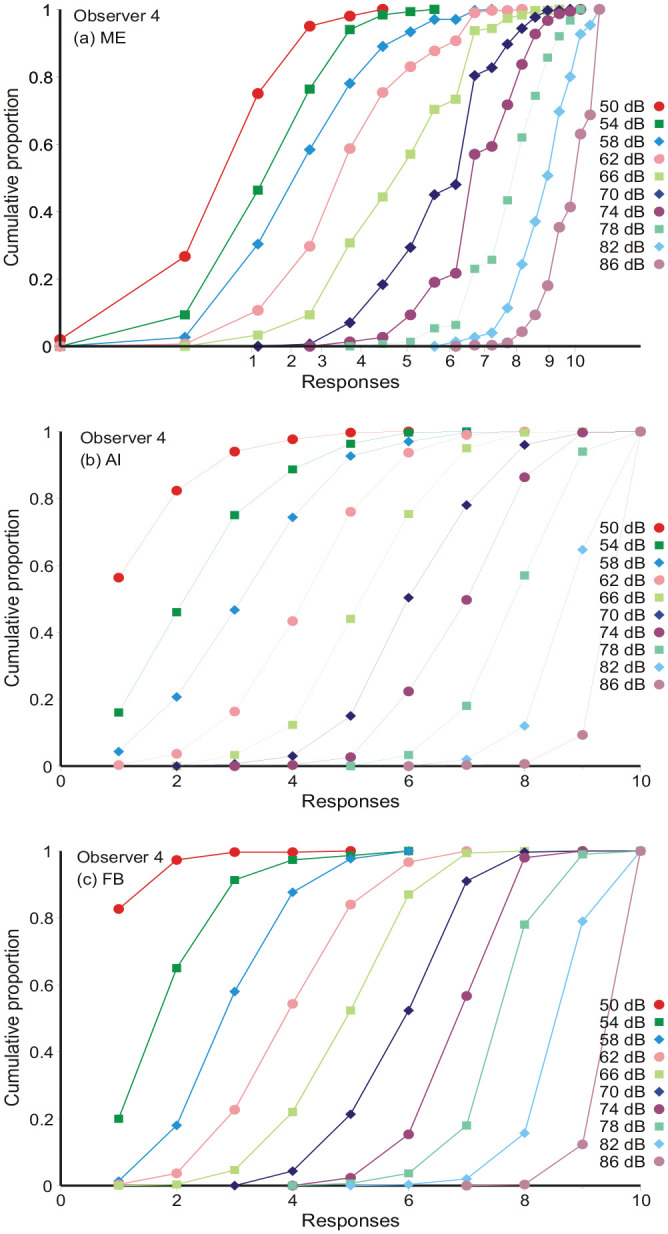
Cumulative distributions of responses from Observer 4. AI and FB responses are plotted against their numerical values on the abscissa. For ME the log_10_ numerical assignments are plotted against an estimate obtained by linear interpolation between the stimulus averages. These stimulus averages are marked on the abscissa of Figure 1a.

Figure 1a to c displays the cumulative distributions of responses for each stimulus and all three conditions for Observer 4.^5^ For AI and FB the responses are naturally plotted against their numerical values on the abscissa. For ME, where the observer chooses his or her own numerical response, the representation is more complicated. First, the numerical responses are transferred to logarithms base 10, and the average log_10_ response calculated for each stimulus. These averages are marked on the abscissa of Figure 1a as “Responses, 1, 2....10.” An approximate value on the stimulus scale, 1...10, is then assigned to each log_10_ estimate by linear interpolation between the stimulus averages. Comparing with AI and FB, the ME data, relative to the stimulus averages, extend below 1 and above 10, whereas AI and FB responses are necessarily constrained. But, setting those extreme responses aside, the ME cumulative distributions are much like AI and FB. Hereafter, log_10_ magnitude estimates are analysed in exactly the same way as AI and FB responses (cf. Braida & Durlach, 1972).

**Figure 2.**
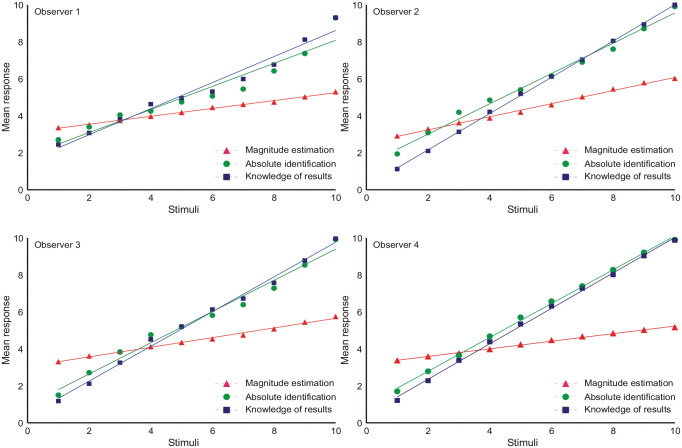
Mean responses as functions of ordinal position, with linear regression lines.

Figure 2 shows the mean response for each stimulus on a scale of 1–10, together with linear regression lines. The departures from linearity are small. The gradients are set out in Table 1.

**Table 1.** Gradients of linear regression in Figure 2.

Observer	ME	AI	FB
1	0.213	0.624	0.705
2	0.355	0.819	0.981
3	0.261	0.844	0.939
4	0.205	0.915	0.958

ME: magnitude estimation; AI: absolute identification; FB: absolute identification with immediate feedback.

Comparing ME, AI, and FB, the gradients for ME are much reduced because the dependent variable is log *N*, not *N*. The gradients for FB are always greater than AI, as one would expect from the provision of knowledge of results. The gradients for these last two are everywhere less than 1, meaning that responses are attracted towards the centre of the stimulus scale. This is the “central tendency of judgment” (Hollingworth, 1909). No similar comparison can be made for ME.

### Subsequent analyses

The analyses that follow examine the autocorrelation of responses, the “bow” effect, and the relation of each response to previous stimuli and responses. These analyses are referred to the ordinal scale of stimuli and responses, 1, . . . 10, as metric. It will help understanding if the results are first sketched informally.

**Figure 3.**
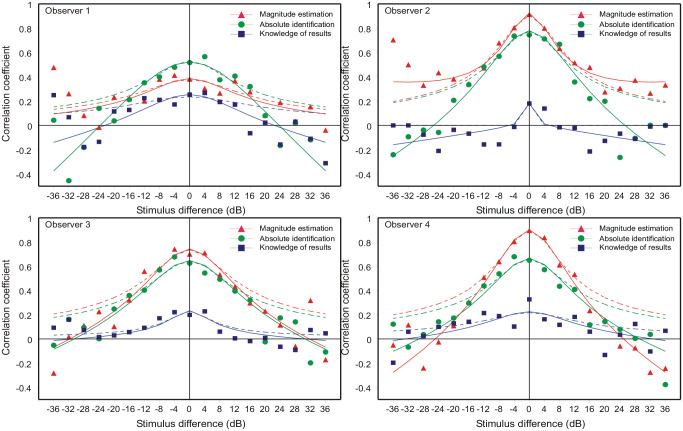
Mean correlation of successive responses as a function of the dB spacing between successive stimuli, *S_n_ – S_n_*_−1_. The continuous curves are Equation 3 (A9 in the online Supplementary Material A) with the α-parameter values in Table 2. The dashed curves are the simpler Equation A4 fit to the range –12 to +12 dB only.

The autocorrelation of responses for AI (Figure 3) replicates the pattern already reported for ME by Jesteadt et al. (1977). This identifies AI and ME as psychologically the same task; the difference between them is simply a matter of how the judgements are expressed. The pattern of autocorrelation signifies, first, that there is no absolute judgement—each stimulus is judged relative to the stimulus and response on the preceding trial (Equation 1)—and, second, there is likewise no absolute assessment of differences or ratios in stimulus magnitude—the adjustment of response from one trial to the next is variable. Each judgement is based on a point of reference that varies from trial to trial by the aggregation of independent increments and, by itself, this aggregation would ordinarily exceed all bounds. A prior expected response (Equation 4), derived from the sequence of stimuli (Equation 7) constrains that otherwise increasing variance. Successive points of reference are still the aggregation of independent increments, but the variance is now limited, and the aggregate distribution is approximately normal (cf. Figure 1).

The model for autocorrelation envisages that sometimes (in AI) an initial assessment may fall outside the range of 1–10. Such an assessment is, of course, forced to “1” or “10,” but that forcing proves essential in accounting for the negative correlations sometimes observed with extreme differences between successive stimuli. This model of autocorrelation is then used to account for the “bow” effect and the sequential effects, which are presented here as consequences of the autocorrelation. The relationships between these different phenomena require some careful calculation; these calculations are placed in the online Supplementary Material.

### Autocorrelation of responses

Figure 3 shows the autocorrelation between successive responses as a function of the dB difference between successive stimuli (*S_n_* − *S_n_*_−1_). For each task there were 3,000 trials organised in blocks of 100. In each block, each stimulus followed each other stimulus (including itself) exactly once. For each combination of successive stimuli (*S_n_* = *S_i_* ∩ *S_n_*_−1_ = *S_j_*), the individual trials were extracted from each block and the subsample of 30 trials analysed separately to deliver a correlation conditional on *S_j_* followed by *S_i_*. Figure 3 plots the mean correlation coefficient for each stimulus difference (*S_i_* − *S_j_*), −36 to 36 dB. Note that the number of individual correlations averaged in each mean varies: it is 10 for zero difference, reducing to 1 at ±36, so that the central means are more precisely determined.

ME repeats the pattern reported by Jesteadt et al. (1977), Baird et al. (1980), and others. When a stimulus is repeated, the mean correlation ranges from 0.38 (Obs 1) to 0.91 (Obs 2), so the second judgement inherits 14% to 82% of its variance from its predecessor. AI shows the same pattern, slightly muted, where the correlations range from 0.52 (Obs 1) to 0.74 (Obs 2). The correlations are, however, much reduced in FB.

It is plain that when a stimulus is repeated, the stimulus and response on trial *n*-1 serves as a reference point for the judgement on trial *n*. Although the correlation is high only for small differences|*S_i_* − *S_j_*|, the use of the stimulus and response on trial *n*-1 as the reference point for trial *n* must be universal, because the observer cannot know until after the stimulus has been presented how big a stimulus difference is to be judged. On the principle that there is no absolute judgement of stimulus quantities, the response on trial *n* is approximately (cf. Laming, 1984, Equation 2.8):

(1) $$R_{n}\;=\;R_{n-1}+B_{n}(S_{n}-S_{n-1})+\varepsilon_{n}$$

where *B_n_* is a random variable and ε_*n*_ an error term of zero mean. The suffix *n* signifies that the values of these variables are specific to trial *n*, but their distributions are common to all trials. Denote the mean of *B_n_* by β, its variance by σ_β_^2^, and the variance of ε_*n*_ by σ_ε_^2^. Equation 1 selects a response on trial *n* that will serve as the reference point for the judgement on trial *n* + 1.^6^

For a suitable choice of parameter values [*R_n_*_−1_, σ_β_^2^, σ_ε_^2^], Equation A4 in Supplementary Material A fits the correlations for |*S_n_* − *S_n-_*_1_| ⩽ 3 well (stimulus difference ⩽12 dB; dashed curves in Figure 3), but for larger stimulus differences the skirts of that equation are all too high. Moreover, the model correlation predicted by Equation A4 is necessarily positive and negative correlations cannot be accommodated at all. This problem occurs at the extreme values of |*S_n_* − *S_n_*_−1_| and arises because initial assessments, *R_n_*, less than 1 or exceeding 10 are forced to “1” or “10.”

**Figure 4.**
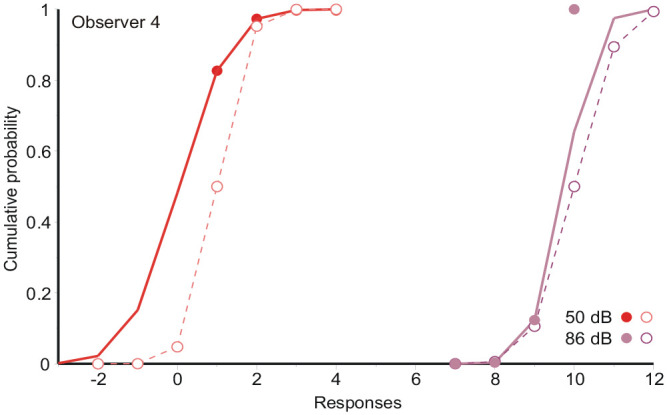
Responses (filled symbols) to the two extreme stimuli in Figure 1c (FB), together with the normal integrals fitted to them, and a conjecture of how the initial assessments (open symbols) might have been distributed. Data from Observer 4.

To illustrate this problem, Figure 4 reproduces the data (***R***_1_, ***R***_10_, filled symbols) from Figure 1c for the two most extreme stimuli, together with conjectures how the initial assessments (*R*_1_, *R*_10_, open symbols) might have been distributed. Initial judgements –2, −1, 0 are forced to “1,” increasing the frequency of “1”s when Stimulus 1 is presented; likewise initial judgements 11 and 12 are forced to “10.” This generates an excess in the observed distribution of ***R***_1_ relative to the prediction from Equation 1 and a deficit in ***R***_10_.

The recorded distribution of ***R***_1_ includes a number of “2,” “3,” “4” responses, so that overshoot of *R*_10_ is most likely when the difference between successive stimuli is large; likewise undershoot of *R*_1_. Accordingly the under/overshoot is modelled with

(2) $$R_{n}\;=\;R_{n}-\alpha (S_{n}-S_{n-1})$$

giving a positive correction when *S_n_* is less than *S_n-_*_1_, greatest when *S_n_* = *S*_1_ (see Figure 4) and a negative correction when *S_n_* is greater than *S_n-_*_1_, greatest when *S_n_* = *S*_10_. This leads (Supplementary Material A, Equation A9) to:

(3) $$\mathrm{Corr}(R_{n},\;R_{n-1})\;=\;\frac{\left[Var(R_{n-1})-\alpha^{2}{\left(S_{n}-S_{n-1}\right)}^{2}\right]}{\sqrt{\mathrm{Var}(R_{n}\mathrm{)Var}(R_{n-1})}}$$

which generates the curved characteristics in Figure 3, with the α-parameter values shown in Table 2.

The formula (Equation A9) depends on the limited range of responses available in AI. In ME there is ostensibly no such limitation; nevertheless, ME shows a similar pattern of correlations, though the estimates of α are smaller.

In FB feedback ostensibly substitutes *S_n-_*_1_ for ***R***_*n-*1_. But reference to *S_n-_*_1_ in place of ***R***_*n-*1_ in Equation 1 seems much less than complete (cf. Holland & Lockhead, 1968, p. 412). Nevertheless, the peak autocorrelation is much reduced and this is reflected in greatly increased estimates of σ_β_^2^ and σ_ε_^2^ in Tables 3a and 3b in Parameter_estimates.doc.^7^

**Table 2.** α-parameter values for the model curves in Figure 3.

Observer	ME	AI	FB
1	0.021	0.253	0.218
2	0^a^	0.236	0.940
3	0.050	0.196	0.160
4	0.045	0.145	0.106

ME: magnitude estimation; AI: absolute identification; FB: absolute identification with immediate feedback.

a The estimate of α^2^ is negative (−.001).

**Table 3.** θ-parameter values for Equations 12 in Figures 9 and 10.

Observer	ME	AI	FB
(a) Partial correlations with preceding stimuli			
1	0.529	0.799	0.890
2	0.875	0.704	0.704
3	0.663	0.671	0.663
4	0.521	0.455	0.834
(b) Partial correlations with preceding responses			
1	0.320	0.480	0.896
2	0.780	0.401	0.780
3	0.198	0.290	0.893
4	0.331	0.256	0.737

ME: magnitude estimation; AI: absolute identification; FB: absolute identification with immediate feedback.

### Prior expectation

Equation 1 generates a sequence of displacements *B_n_*(*S_n_ – S_n_*_−1_) +ε_*n*_ of the point of reference, *R_n-_*_1_, of which the sum of squares, Σ_*n*_(*B_n_*(*S_n_ – S_n_*_−1_) +ε_*n*_)^2^ would, without modification, grow beyond all bounds. Suppose that observers have some idea of what kind of response would be appropriate, and let *R*_π_ be that prior expectation. The actual response is then a weighted average (cf. Laming, 1999, Equation 4):

(4) $$R_{n}\;=\theta [R_{n-1}+B_{n}(S_{n}-\;\;S_{n-1})+\varepsilon_{n}]\;+(1-\theta )R_{\pi }$$

where (1 − θ) is the weight accorded to the prior expectation. The point of reference for trial *n* is now θ*R_n_*_−1_ + (1-θ)*R*_π_ and the cumulative variance becomes Σ_*n*_θ^2n^(*B_n_*(*S_n_ – S_n_*_−1_) +ε_*n*_)^2^, which is finite for θ < 1. Rephrasing Equation 4:

(5) $$\left(R_{n}-\;R_{\pi }\right)=\theta [\left(R_{n-1}-\;R_{\pi }\right)+B_{n}(S_{n}-\;\;S_{n-1})+\varepsilon_{n}]\;$$

and responses are hereafter referenced to θ(*R_n_*_−1_ – *R*_π_).

The prior expectation *R*__π__ must itself be derived from previous observation of the stimuli. Rewrite Equation 5 as

(6) $$(R_{n}-\;R_{\pi ,n})=\theta [(R_{n-1}-\;R_{\pi ,n-1})+B_{n}(S_{n}-\;\;S_{n-1})+\varepsilon_{n}]\;$$

where^8^

(7) $$R_{\pi ,n}=\theta R_{\pi ,n-1}\;+(1-\theta )S_{n-1}$$

Prior expectation thereby adjusts to the sequence of stimuli.

Equation 6 makes the point of reference for the judgement of *S_n_* the sum of many small adjustments and therefore, at least approximately, normal (cf. Figure 1), but now of finite variance. Herein lies the source of the frequent normal variation in many signal detection and judgement tasks.

### The “bow” effect

**Figure 5.**
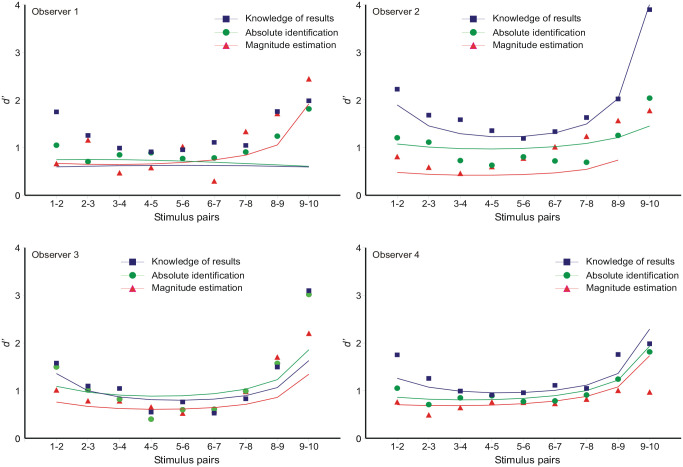
The “bow” effect: estimates of *d*′ between adjacent stimuli. The model curves are generated by inserting the variances from Equation 9 (cf. Figure 6) into Equation 8.

**Figure 6.**
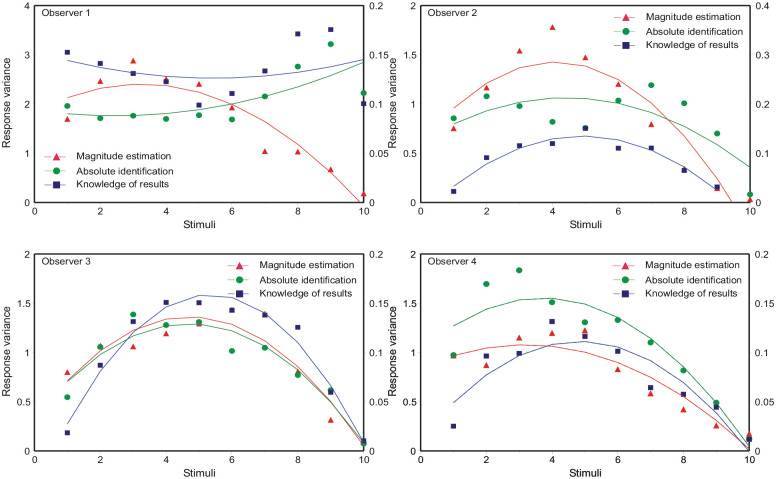
Response variance as a function of ordinal position in the stimulus set. The model curves are Equation 9 (B9 in the online Supplementary Material B), with the parameter values in Tables 6a, b, c in Parameter_estimates.doc.

Estimates of *d*′ for the discrimination between adjacent stimuli (*S_i_* vs. *S_i_* __+__ _1_) show a depression in the middle of the stimulus set (Luce et al., 1982; Weber et al., 1977). Figure 5 shows *d*′s calculated from the aggregate data in Figure 1 and the other observers. A normal probability integral was fit to the responses to each stimulus; then *d*′ for the discrimination between *S_i_* and *S_i_* __+__ _1_ was estimated as

(8) $${d^{\prime }}_{i,i+1}=\;\left(\mu_{i+1}-\;\mu_{i}\right)/\sqrt{(\sigma_{i}^{2}+\sigma_{i+1}^{2})/2}$$

where µ_*i*_, µ_*i*_ _+_ _1_ are the adjacent means and σ_*i*_^2^, σ_*i*_ _+_ _1_^2^ the corresponding variances. Figure 2 shows the mean responses to be approximately linear with respect to the ordinal scale of the stimuli, so that the “bow” effect must result from an increase in response variance in the centre of the stimulus set. This is confirmed in Figure 6, which shows the response variances conditional on each stimulus. An expression for these response variances is derived in Supplementary Material B.

The data in Figure 6 are asymmetric, while Equation B8 is symmetric about $\bar{S}$ . This asymmetry can be corrected, approximately, by writing *R*_π_ for $\bar{S}$ , giving (Supplementary Material B, Equation B9):

(9) $$\begin{array}{c} \mathrm{Var}(R_{i}-R_{\pi })=N^{-1}\left(2\theta -\theta^{2}\right)\sum_{j}\mathrm{Var}(R_{j}-R_{\pi })+\\ \left(\theta^{2}\sigma_{\beta }^{2}-\theta \alpha^{2}\right)\left[{\left(S_{i}-R_{\pi }\right)}^{2}+8.25\right]+\\ \theta^{2}\mathrm{Var}(\varepsilon_{n}) \end{array}$$

This equation generates the asymmetric curves in Figure 6, with the parameter values in Tables 6a, b, c in Parameter_estimates.doc. Entering the variances calculated from Equation 9 into Equation 8, with unit difference of mean, then generates the predictions for *d*′ in Figure 5.

Observer 1 in Figure 6 shows response variances that are slightly less in the centre of the stimulus set, except for the extreme stimulus (86 dB) for which the variance is much reduced. Equation B8 in the Supplementary Material delivers response variances peaking in the centre of the stimulus set *provided* $(\theta^{2}\sigma_{\beta }^{2}-\theta \alpha^{2})<0$ . Observer 1 is an exception.

### Sequential effects^9^

**Figure 7.**
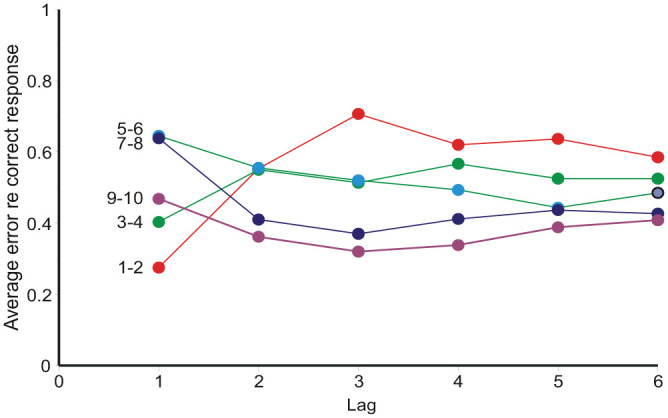
Average “error,” ***R***_*n*_–*S_n_*, conditional on each adjacent pair of stimuli on the trial 1, 2 ... 6 places previous (Condition AI, Observer 4).

**Figure 8.**
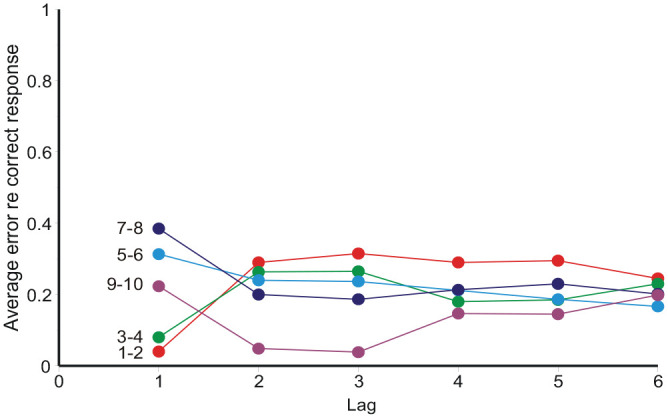
Average “error,” ***R***_*_n_*_–*S_n_*, conditional on each adjacent pair of stimuli on the trial 1, 2 ... 6 places previous (Condition FB, Observer 4).

Category judgements are related to the preceding stimuli and responses. Holland and Lockhead (1968, Figure 3) plotted the “error,” ***R***_*n*_–*S_n_*, conditional on each adjacent pair of stimuli on the trial 1, 2 .... 6 places previous. The difference (***R***_*n*_–*S_n_*) assimilates to *S_n_*_−1_ (positive correlation), but contrasts (negative correlation) with *S_n_*_−2_, *S_n_*_−3_, *S_n_*_−4_, etc. This pattern has been observed both with and without feedback and equally in relation to previous responses (Ward & Lockhead, 1970, 1971). It has been widely reported by other investigators as well (see Stewart et al., 2005, p. 883). Figures 7 and 8 display a like analysis of the present data; it exhibits the same pattern, except that this observer shows a systematic overestimation; mean ***R***_*n*_–*S_n_* is everywhere positive.

However, a simple reading of Equation 1 says that ***R***_*n*_ should correlate *negatively* with *S_n_*_−1_. The analyses in Figures 7 and 8 (equally in Ward & Lockhead, 1970, 1971) are equivalent to calculating the *total* regression of ***R***_*n*_–*S_n_* on *S_n_*_−*k*_, separately for each *k*. The response ***R***_*n*_ correlates positively with ***R***_*n*-1_ (Equation 1) and ***R***_*n*-1_, of course, correlates positively with *S_n_*_−1_. So, in the absence of reference to ***R***_*n*-1_, ***R***_*n*_ will appear to correlate positively with *S_n_*_−1_. But the inclusion of ***R***_*n*-1_ in Equation 1 generates a quite different perspective. Any analysis of the relation of ***R***_*n*_ to preceding stimuli *must* take preceding responses into account.

The *total* regression of ***R***_*n*_–*S_n_* on *S_n_*_−*k*_ in Figures 7 and 8 is also difficult to interpret, because the autocorrelation of responses in Figure 3 means that each ***R***_*n*-*k*_–*S_n_*_−*k*_ interacts with *S_n_*_−*k*-1_ and that interaction propagates through succeeding trials. A clear understanding requires a multiple regression of ***R***_*n*_ on preceding stimuli and responses (cf. Jesteadt et al., 1977):

(10) $$\begin{array}{l} R_{n}=c+a_{0}S_{n}+a_{1}S_{n}{}_{-1}+a_{2}S_{n}{}_{-2}+\\ \;\;\;\;\;\;\;a_{3}S_{n}{}_{-3}¼+b_{1}R_{n}{}_{-1}+b_{2}R_{n}{}_{-2}+b_{3}R_{n}{}_{-3}¼+\varepsilon_{n} \end{array}$$

**Figure 9.**
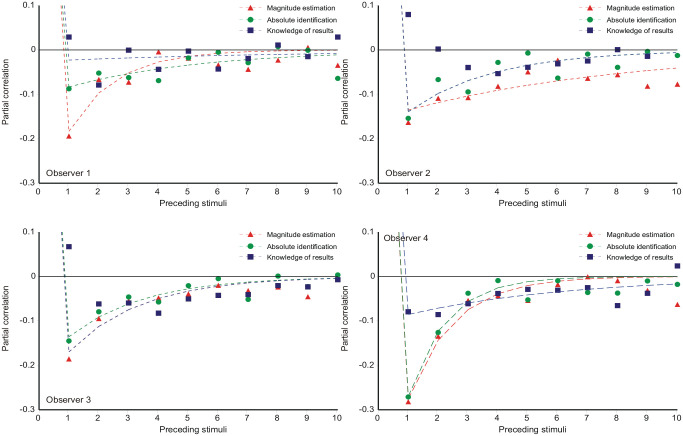
Partial correlation coefficients between ***R***_*_n_*_ and *S_n-k_, k* = 1, . . . 10. The curves are Equation 11 multiplied by a scaling factor (Equation C10), with the parameter values in Tables 9a, b, c in Parameter_estimates.doc.

**Figure 10.**
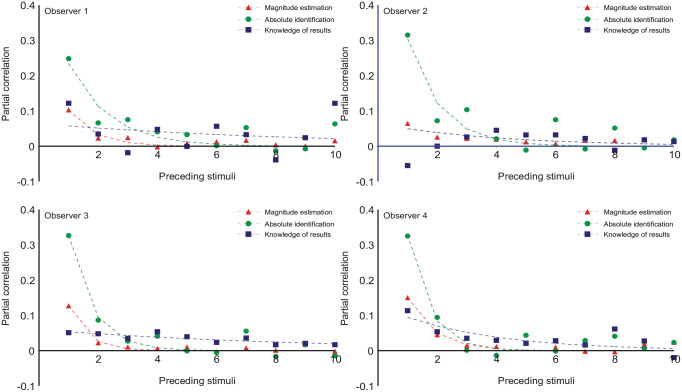
Partial correlation coefficients between ***R***_*n*_ and ***R***_*n-k*_, *k* = 1, . . . 10. The coefficients and curves for ME are shown at 1/10 scale. The curves are Equation 12 with the parameter values in Tables 10a, b in Parameter_estimates.doc. The θ-parameter (relation of prior expectation to preceding stimuli; Equation 7) is additionally reproduced in Table 3.

In Equation 10, *c* is a constant (with the effect of centring all variables to zero mean), ε_*n*_ an error term, and the *a_k_* and *b_j_* regression coefficients are estimated by minimisation of the sum of squares. The *a_k_* estimate Cov(***R***_*n*_, *S_n-k_*)/Var(*S_n-k_*), and multiplying by √[Var(*S_n-k_*)/Var(***R***_*n*_)] converts them into the partial correlation coefficients between ***R***_*n*_ and *S_n-k_, k* = 1, 2, ..., shown in Figure 9 (the coefficients *a*_0_ exceed 0.9 and are not shown in the figure as a matter of economy. They, nevertheless, enter into the fit of the model in Supplementary Material C). Likewise, the *b_k_* estimate Cov(***R***_*n*_, ***R***_*n-k*_)/Var(***R***_*n-k*_), and multiplying by √[Var(***R***_*n-k*_)/Var(***R***_*n*_)] converts them into the partial correlation coefficients between ***R***_*n*_ and ***R***_*n-k*_, *k* = 1, 2, ..., as shown in Figure 10. Multiplication by √[Var(*S_n-k_*)/Var(***R***_*n*_)] and √[Var(***R***_*n-k*_)/Var(***R***_*n*_)], respectively, brings the partial correlation coefficients from different conditions into registration with each other, irrespective of the different variances of the responses, and enables a like analysis of ME to be included. The presentation sequences were randomly ordered, so that the different *S_n-k_* are, at least approximately, independent, and to that extent the partial correlations approximate total correlations.

### Partial correlations with previous stimuli

The equations

(11) $$\begin{array}{c} a_{0}=\theta \beta \\ a_{1}=\;(1-\theta )(1-\theta \beta )\\ a_{i}=\theta a_{i}{}_{-1} \end{array}\}$$

(Supplementary Material C, Equation C5) generate the curves in Figure 9 with the parameter values in Tables 9a, b, c in Parameter_estimates.doc. The θ-parameter (relation of prior expectation to preceding stimuli; Equation 7 is additionally reproduced in Table 3.

### Partial correlations with previous responses

Figure 10 shows the partial correlations of ***R***_*n*_ with previous responses, ***R***_*n-k*_, *k* = 1, . . . 10. The fitted functions are simple negative exponentials:

(12) $$b_{k}=b_{1}\theta^{k}{}^{-1}$$

(Supplementary Material C, Equation C11) with parameter values in Tables 10a, b in Parameter_estimates.doc. The partial correlations for ME and AI in both Figures 9 and 10 show a satisfactory fit to Equations 11 and 12, respectively, which, in turn, describe an exponentially decreasing trend. But FB is different because of the feedback. Ostensibly, this substitutes *S_n_*_−1_ for ***R***_*n*-1_ in Equation 1, but this is too simple, as there would then be no sequential effects. The chief difference is an enlarged value of θ for Observers 1 and 4 in Figure 9 (the data for Observers 2 and 3, FB, do not fit the model) and for all observers in Figure 10 (see Table 3). Assuming feedback contributes directly to prior expectation (Equation 7) the precision of that expectation is much increased and likewise its contribution to the choice of response (cf. Equation 4).

Both Equations 11 and 12 are consistent with an interaction between ***R***_*n*_ and ***R***_*n*-1_ that propagates through successive trials to generate partial correlations with previous responses, there being no additional relationship with, say, ***R***_*n*-2_. This is consistent with the finding of Jesteadt et al. (1977, Table 1), who found no increase in multiple correlation in their regression analysis from the inclusion of stimuli and responses more remote than *S_n_*_−1_, ***R***_*n*-1_.

Jesteadt et al. (1977) intuited that the entire pattern of sequential interactions was generated by the one interaction between ***R***_*n*_ and ***R***_*n*-1_, propagated through succeeding trials. Repeating their analysis in a slightly modified form, Table 4 (Observer 4, but see also Tables 4a, b, c, d in Parameter_estimates.doc) lists the *square* of the multiple correlation coefficient (i.e., *r*^2^) for each additional *pair* of variables (*S_n_*_−*k*_, ***R***_*n*-*k*_) included in the regression (Equation 10), together with the increment in *r*^2^, which is proportional to the reduction in the residual sum of squares. Most of the multiple correlation is, of course, contributed by *S_n_; S_n_*_−1_ and ***R***_*n*-1_ then effect a principal reduction in the sum of squares; but further reductions (Δ*r*^2^) consequent on the inclusion of subsequent pairs (*S_n_*_−*k*_ and ***R***_*n*- *k*_) are smaller than that principal reduction by an order of magnitude.

**Table 4.** Squares of the multiple correlation coefficient (*r*^2^) for different numbers of terms included in Equation 10 (Observer 4).

Regression	ME		AI		FB	
variables	*r* ^2^	Δ*r*^2^	*r* ^2^	Δ*r*^2^	*r* ^2^	Δ*r*^2^
*S_n_*	0.8169		0.8597		0.909	
*S_n_*_−1_, ***R***_*n*-1_	0.8509	0.034	0.88	0.0203	0.9116	0.0026
*S_n_*_−2_, ***R***_*n*-2_	0.8555	0.0046	0.8826	0.0026	0.9131	0.0015
*S_n_*_−3_, ***R***_*n*-3_	0.8566	0.0011	0.8839	0.0013	0.914	0.0009
*S_n_*_−4_, ***R***_*n*-4_	0.8574	0.0008	0.8843	0.0004	0.9142	0.0002
*S_n_*_−5_, ***R***_*n*-5_	0.8582	0.0008	0.8847	0.0004	0.9143	1E-04

ME: magnitude estimation; AI: absolute identification; FB: absolute identification with immediate feedback.

So, to a good first approximation, the pattern of sequential relationships is driven by the interaction between ***R***_*n*_ and (*S_n_*_−1_, ***R***_*n*-1_), propagated through successive trials. However, it should not pass without notice that many of the incremental reductions in Table 4 are highly significant (*p* < .0001) up to about *n*-5. The mechanism of sequential interaction is not simply linear.

## Discussion

Responses to the same stimulus repeated on successive trials correlate about +0.38 (Obs 1) to 0.91 (Obs 2), (AI, Figure 3). It must be that the first of those responses is used as a point of reference for the second—there is no absolute judgement of the second stimulus. This idea was first proposed by Holland and Lockhead (1968, p. 412):

In an absolute judgment task with feedback provided, it is proposed that Ss use the remembered magnitude of the stimulus on the preceding trial, and the numeric value of the feedback for that stimulus, as a standard for a comparative judgment of the presented stimulus.

If the stimuli on successive trials differ, the correlation is less, even negative for large differences. But it must still be that the response on one trial is used as a point of reference for the next, because the observer cannot know in advance how that second stimulus will differ from the first. The correlation is less because the extrapolation from one response to the next is variable—there is no absolute judgement of differences or ratios between stimuli. The pattern of autocorrelation in Figure 3 and elsewhere has a profound effect on our understanding of category judgement.

Now, to answer the questions set out in the introduction:

1. AI without feedback shows the same pattern of autocorrelation as ME. The process of judgement must be the same in both tasks. Minor differences arise because the observer in AI knows that there are (in this experiment) only 10 different stimuli, so that a stimulus that appears to be No. 0 or 11 forces a reappraisal of the framework of judgement.
2. If AI without feedback is a relative judgement, then so also is AI *with* feedback. Feedback enters only *after* a judgement has been output to modify the point of reference for the following trial. It ostensibly substitutes *S_n_*_−1_ for *R_n_*_−1_ in Equation 1, and

(13) $$\mathrm{Cov}\;(R_{n},\;S_{n-1})\;=\;0$$

in Equation 2, because *S_n_*_−1_ is fixed. The pattern of autocorrelation in Figure 3 is consequently much attenuated. This is important because Stewart et al. (2005) and subsequent contributors to that debate have not ordinarily distinguished between AI with, and without, feedback.

Stewart et al. (2005) proposed a determinate process to link the response on one trial to that on the next—it amounts to an *absolute judgement* of stimulus differences/ratios (cf. the response–ratio hypothesis, Luce & Green, 1974). Guest et al. (2016) compared conventional AI of 8 lengths of line (Expt 1) with a similar AI of the absolute *difference* between successive lengths (Expt 2). Their two sets of results are not comparable. In particular, a repeated stimulus gives 65% correct responses in conventional AI (Expt 1), but only 35% in the AI of differences (Expt 2). This may fairly address the particular model proposed by Stewart et al. (2005), with determinate linkage from one trial to the next; but neither of these studies accommodates the pattern of autocorrelation in Figure 3.

The debate, relative judgement versus AI, has coalesced on the specific proposal of an *absolute judgement* of stimulus differences/ratios by Stewart et al. (2005). The results here point to a *third* idea, not previously considered (except Laming, 1984, 1997). Each stimulus in an experiment is compared with its predecessor, greater, less than, or about the same. The *variability* of that comparison increases with the difference in magnitude between the stimuli, so the assessment of a stimulus far removed from its predecessor is very uncertain. This idea accommodates the pattern of autocorrelation in Figure 3, which, in turn, proves incompatible with an *absolute* judgement of stimulus differences/ratios. It provides a radical new insight into category judgement. The rest of this discussion explores what changes are now needed to present understanding.

### Thurstone’s “Law of Comparative Judgment”

Thurstone (1927b) proposed that a stimulus, as perceived by the observer, is intrinsically variable. This would explain why psychophysical judgements, especially discriminations between one stimulus and another, are so variable. Taking that variability to be normally distributed, Thurstone sought to establish a stimulus scale from experimental data. Thurstone (1927a) distinguished five different formulations of this idea, of which “Case V” is much better known these days as the normal, equal variance, signal-detection model.

Torgerson (1958) developed Thurstone’s “Case V” to accommodate a linear array of stimuli. There are *N* normal distributions of means µ_*i*_ and common variance σ^2^,

(14) $$f_{i}\left(x\right)\;=\;{\left(2\pi \sigma^{2}\right)}^{-1/2}\exp\{-{\left(x-\mu_{i}\right)}^{2}/2\sigma^{2}\},i=\;1,\ldots ,N$$

one for each stimulus value, *i* = 1, . . . ., *N*, and *N*-1 criteria, *c_j_, j* = 1, . . . . , *N*-1. The probability of response *j* given stimulus *i, P*(*R_j_*|*S_i_*), is represented by the proportion of the *S_i_* distribution that lies between criteria *c_j_*_−1_ and *c_j_*:

(15) $$P(R_{j}|S_{i})=\int_{c_{j-1}}^{c_{j}}f_{i}(x)\;dx$$

The standard deviation is commonly fixed at 1 and the mean of the smallest stimulus at 0; all the other stimulus means and criterion values are then estimated from the data. This implicitly establishes an interval scale of the stimulus continuum in units of the standard deviation and provides a scaffold for the AI of whatever stimulus is presented, subject, of course, to the error σ^2^ (Braida & Durlach, 1972; Durlach & Braida, 1969; Luce et al., 1976; Treisman, 1985).

Other ideas have been proposed to serve the same function. Some musicians, though not all, can identify an auditory frequency to an accuracy of a quarter tone (about 3%). In general they do not know how they do it, but it appears to be a consequence, rather than a precursor, of their musical training. “Perfect pitch” extends only over the range of the piano keyboard, and Bachem (1954) confesses to making the occasional error of an octave. To my mind, that is absolute judgement. Nosofsky (1986), G. D. A. Brown, Neath, and Chater (2007) and Kent and Lamberts (2005) among others, have proposed exemplar models based on the storage of magnitudes in memory. Stewart et al. (2005, Table 2) have tabulated the principal phenomena of AI and FB that these, and other models, are able to accommodate (though not including autocorrelation).

The present data do not speak of any of those ideas. However, if the judgement of the difference between successive stimuli was absolute, then the correlation between successive responses would be independent of the difference between successive stimuli. The pattern of autocorrelation in Figure 3 tells us that the assessment of the difference between successive stimuli is itself relative (Equation 1; Laming, 1984).

Seeking the simplest account of the present data, the “bow” effect and the sequential interactions are related directly to the pattern of autocorrelation. It is necessary to introduce initial “Off the scale” assessments; it is not possible to identify such assessments in the recorded data, but—try AI for yourself! It is also necessary to diminish the weight of preceding trials exponentially—the judgement process is, at least approximately, Markov. But structures in memory supporting an absolute comparison are not needed.

As Equation 14 stands, there is no autocorrelation, nor sequential effects. So, what needs to change?

The roles of the stimuli and criteria need to be interchanged (cf. Lockhead, 2004). There will be *N* stimuli, µ_*i*_, *i* = 1, . . . ., *N*, as before, but no directly associated variability. Instead, successive stimuli are categorised against a continually evolving point of reference. Stimulus *S_i_* generates response *R_j_* when that momentary point of reference is equivalent to a criterion lying between *c_j_*_−1_ and *c_j_* (cf. Equation 15). This interchange of stimuli and criteria leaves the model probabilities calculated from Equation 14 unchanged, but the variability is no longer a property of the stimulus—it is internal to the observer.

If the variability of judgement is no longer intrinsic to the stimulus, it cannot be used to generate a scale of the sensory continuum. Instead, the only metric for analysis is the ordinal scale of the stimulus set and the analyses above have been formulated in this ordinal metric. At the same time, the point of reference evolves by small additive increments from trial to trial (Equation 5) and this explains why experimental data are so well accommodated by normal distributions (Figure 1).

### Autocorrelation

The striking pattern of autocorrelation of log magnitude estimates was discovered by Jesteadt et al. (1977) in the course of a routine regression analysis of log *N_n_* on log *S_n_*, log *S_n-i_*, and log *N_n-i_*, intended to test the response–ratio hypothesis of Luce and Green (1974). Green et al. (1977) then showed that the same pattern appeared in magnitude production of loudness (1 kHz tones again) as well as in ME. Luce and Green (1978) showed that the maximum degree of correlation was reduced (to +0.5 to 0.6) in an experiment where the frequency of the tone changed (1 to 4 kHz) from one trial to another. Baird et al. (1980) reported a similar pattern of correlation for the area of random geometric shapes and an attenuated pattern (maximum correlation +0.37) for cross-modality matching of loudness to area. Further correlations have been contributed by Ward (1979) for the separation between two dots on a screen and for the matching of duration to separation. In addition, Siegel (1972) reported that accuracy in the identification of auditory frequency was much increased for a repeated stimulus, a finding that was replicated by Stewart et al. (2005, Fig. 25). But this pattern of autocorrelation has not previously been reported for AI.

### “Off-the-scale” assessments

The manner of selecting the next response (Equation 1) means that some initial assessments will be “off the scale,” “0” or “−1” or “10,” “11” or “12.” Although this mostly affects responses to the extreme stimuli (1 and 10), the cumulative data in Figure 1 suggest that it involves other adjacent stimuli as well and, relative to the stimulus averages, the ME data in Figure 1a extend below 1 and above 10. Such responses are, of course, forced in AI to “1” or “10.” That forcing needs to be taken into account, because the pattern of autocorrelation in Figure 3 relates to the responses actually observed, not the (model) responses that constitute the initial assessment. Happily the model of forcing (Equation 2) leads to a simple modification of the model of autocorrelation (Equation A9, A10) and accommodates the negative correlations that sometimes occur following extreme differences of stimulus value. In principle, there should be no “off the scale” responses in ME, but individual negative correlations can be found in the figures published by Green et al. (1977); Jesteadt et al. (1977); Luce and Green (1978) and Baird et al. (1980). In the present experiment, the pattern of autocorrelation for ME is more sharply focused even than for AI and some of the ME correlations are negative.

### The “bow” effect

Analyses of the “bow” effect are commonly based on Equation 14, which forces all standard deviations to be the same; the “bow” effect then subsists in a contraction of the estimated means towards the centre of the stimulus scale. But, relative to the ordinal spacing of the stimuli, the response variances are not equal (Figure 6). The relation of mean response to stimulus location is approximately linear (Figure 2), so the “bow” effect must be consequent on that increased response variance in the middle of the stimulus set. The model of autocorrelation generates a model for these variances (Equation B8), which, in turn, generates a model for the “bow” effect (Figure 5) by substitution into Equation 8.

The “bow” effect (Equation B8) is parameter-dependent. If, for example, α, the adjustment for under/overshoot in Equation 2, was 0, the bow would be inverted. Observer 1 in Figure 6 appears to present such an example, except that the variance of responses to the most extreme stimulus (86 dB) is much reduced.

The response variances in Figure 6 are asymmetric—variance is less in the upper half of the scale and this leads to increased values of *d*′. A similar asymmetry can be found in the data of Luce et al. (1982) and Weber et al. (1977). Several authors have drawn attention to the role of the extreme stimuli, suggesting they serve as “anchors” (Berliner & Durlach, 1973; Durlach & Braida, 1969; Weber et al., 1977). “Off-the-scale” assessments enable those extreme stimuli to be identified with greater accuracy than other stimuli. Stewart et al. (2005) invoke a “limited decision capacity,” which is greatest for the central stimuli; putting that another way, there is less opportunity for error at the ends of the stimulus range.

### Sequential effects

Initially sequential effects present a puzzle: responses appear to correlate positively with the preceding stimulus, but negatively with all earlier stimuli. It happens so because *R_n_* correlates positively with *R_n_*_−1_, and *R_n_*_−1_ correlates positively with *S_n_*_−1_. If *R_n_*_−1_ is left out of the reckoning, there remains a positive correlation between *R_n_* and *S_n_*_−1_ (Ward & Lockhead, 1970, 1971; Figures 7 and 8 here). Stewart et al. (2005, Equation 8) invoke a confusion of the current stimulus difference (*S_n_* − *S_n_*_−1_) with representations of other previous differences, whereupon a suitable choice of parameters gives assimilation to *S_n_*_−1_ and contrast with all previous stimuli. But if sequential relationships are analysed with a regression equation containing both stimulus and response terms (Equation 10), the partial correlation coefficients for ME and AI are all negative (except for the trial stimulus) and show a geometric decreasing trend (Figures 9 and 10), suggesting a Markov process.

Jesteadt et al. (1977) intuited exactly this of their ME data. Table 4 repeats their regression analysis in a slightly modified form to the same effect. Regression on (*S_n_*_−1_, ***R***_*n*-1_) jointly produces a small reduction in the residual sum of squares, but the reductions produced by additional terms (*S_n_*_−*k*_, ***R***_*n*-*k*_), *k* > 1, are smaller by an order of magnitude. To a first approximation the pattern of sequential relationships is driven by the interaction between ***R***_*n*_ and (*S_n_*_−1_, ***R***_*n*-1_) alone, propagated through successive trials.

### Stimulus range

If Thurstone’s *Law of Comparative Judgment* (Equation 14) applied, then, increasing the range covered by a fixed number of stimuli, the variance σ^2^ remaining constant, should lead to continually improved resolution; but it does not happen so. Braida and Durlach (1972, Expt. 4) compared AI with feedback for 10 500-ms bursts of 1,000 Hz tones spaced, in different conditions, at 0.25, 0.5, 1, 2, 3, 4, 5, and 6-dB intervals with the highest level always at 86 dB. There were about 1,875 trials per condition for each of three observers. If the variance (σ^2^) in Equation 14 is estimated from the aggregate data, but separately for each stimulus spacing, it increases in proportion to the square of that spacing, as

(16) $${\hat{\sigma }}^{2}\equiv 1.52+0.49\times {\left(\mathrm{spacing}\;\mathrm{in}\;\mathrm{dB}\right)}^{2}$$

(see Braida & Durlach, 1972, Figure 4c; Laming, 2010, Figure 11, p. 762).

Braida and Durlach (1972, p. 484) proposed a “memory” variance, increasing in proportion to the square of the stimulus range [i.e., 0.49 × (spacing in dB)^2^], so that the dispersion of identifications increases in proportion to the spacing of the stimuli. For small ranges dispersion is additionally increased (1.52) by intrinsic variability attributed to the stimulus (Equation 14). The analysis here retains the “memory” variance (Equation 4)—dispersion increasing in proportion to stimulus spacing—but abandons Thurstone’s intrinsic variability. Instead, at small ranges dispersion is increased by a presumed variability between different sections of the data (σ_ε_^2^ in Equation 4).

**Figure 11.**
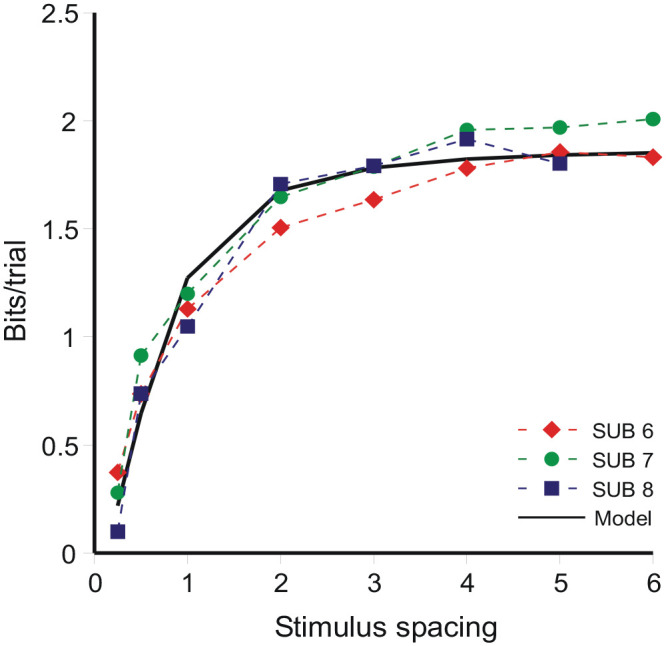
Information transmitted per trial for three observers in Braida and Durlach’s (1972) Expt 4. The model curve is Equation 17.

The limit to resolution as a function of stimulus spacing is shown in Figure 11 as information transmitted in bits per trial. It increases to a limit, as spacing increases, of about 2 bits/trial, equivalent to the identification of no more than four stimuli without error. Information transmitted increases as the inverse of the variance relative to the stimulus spacing. Simplifying (Equation B9),

(17) $$\mathrm{Var}\left(R_{n}\right)\;\equiv \;A\mathrm{Var}(S_{n})+B$$

where

(18) $$\begin{array}{c} A=\left(\theta^{2}\sigma_{\beta }^{2}-\theta \alpha^{2}\right),\\ B=N^{-1}\left[2\theta \sum_{j}\mathrm{Var}(R_{j})-\theta^{2}\sum_{j}\mathrm{Var}(R_{j})\right]\\ +8.25\left(\theta^{2}\sigma_{\beta }^{2}-\theta \alpha^{2}\right)+\theta^{2}\mathrm{Var}(\varepsilon_{n}) \end{array}$$

and Var(*S_n_*) (= 8.25) is calculated with respect to the ordinal metric of the stimulus set. Relative to the stimulus spacing in dB, this becomes

(19) $$\mathrm{Var}\left(R_{n}\right)/\mathrm{Var}(S_{n})\;\equiv \;A+B/\left(8.25\times {\mathrm{spacing}\;\mathrm{in}\;\mathrm{dB}}^{2}\right)$$

and is the curve fitted in Figure 11 with *A* = 0.53 and *B* = 0.25. Figure 11 displays a configuration very similar to Braida and Durlach’s Figure 4d (1972, p. 491), which presents total sensitivity aggregated over the stimulus range (see also Stewart et al., 2005, Figure 11).

Notwithstanding Braida and Durlach (1972, Expt. 4), it is still sometimes claimed that “measures of discriminability depend on stimulus range” (Gravetter & Lockhead, 1973; Lockhead & Hinson, 1986, p. 53). Lockhead (2004) made the very pertinent point that the intensity of a 1-kHz tone (or the brightness of a light) is but one attribute of a complex stimulus. As a matter of convenience, the intensity of tone or brightness of light is abstracted from the rest of the complex, while the rest is ignored in the analysis of the data. What applies to the physical structure of the stimulus applies also to its temporal structure—specifically the sequence of stimuli and the range of values they span. Lockhead and Hinson compared the three different sets of stimulus magnitudes shown in Table 5 using 0.5-s, 1-kHz tones and both AI and FB.

**Table 5.** Levels of 1-kHz tones in the experiment by Lockhead and Hinson (1986).

Condition	Stimulus magnitudes (dB)				
Low-spread	54		60	62	
Normal		58	60	62	
High-spread		58	60		66

The accuracy of discrimination between 60 and 62 dB was impaired by the 54-dB stimulus in the low-spread condition, compared with the normal; likewise the discrimination between 58 and 60 dB in the high-spread condition. But the assessment of 60 dB, whatever the preceding stimulus, is subject to some variability, and that variability is greater when 54, rather than 58 dB, precedes (Equation 1). This increased variability impairs the resolution of 60 from 62 dB.

Envisage an experiment with seven different tones from 54 to 66 dB at intervals of 2 dB. Identify the (*S_n_*_−1_, *S_n_*) pairs corresponding to the successive pairs of stimuli in Lockhead and Hinson’s experiment and analyse specifically the responses to those *S_n_*. Reference to Figure 3 shows that the correlation following a 54 or 66 dB *S_n_*_−1_ will be less, and the variance transmitted from trial *n*-1 to trial *n* greater, than with a 58 or 62 dB *S_n_*_−1_. In short, the impairment of pair-wise discrimination contingent on the remoteness of the preceding stimulus (cf. the “bow” effect in Figure 5) is implicit in the present experiment, which is consistent with Lockhead and Hinson’s findings. S. D. Brown et al. (2009) have replicated Lockhead and Hinson’s experiment with the same outcome.

### Response times

S. D. Brown et al. (2009) recorded response latencies and Lacouture (1997) explicitly looked for the “bow” effect and sequential relationships in response time data. In fact, Lacouture replicated Laming (1968, Expt. 6), though with 10 stimuli rather than only 5. Laming presented a series of 800 signals to each of 24 subjects with a 2-min break after every 200 signals. The signals were lengths of line (as Lacouture) at a geometric spacing of 1.41. The signals were mapped in order of length onto five response keys operated with the digits of the right hand. The following observation seems especially relevant:

Some subjects showed at times a certain confusion of the signals, which took the form of a transposition of one or more signals to adjacent responses [adjacent on the keypad], up or down the scale. These transpositions were temporary, though they might last for 50 responses and might occur several times during the same experimental session. The transposition of the signals was rarely complete and some correct responses occurred during these periods. In some cases it is known that such correct responses were regarded by the subject as errors [“damn”]. Laming (1968, p. 72)

### Limit to information transmitted

It has been known since Hake and Garner (1951) and Garner and Hake (1951) that observers are unable to distinguish more than five stimuli on a single continuum without error. This finding has been replicated with many different stimulus attributes (see Laming, 1984, Table 1; Stewart et al., 2005, Table 1 for tabulation of relevant studies) and the limit of 5 is exceeded only when there is a physical reference enabling the continuum to be, as it were, divided into two independent ranges (Hake & Garner, 1951, is one example), with up to five stimuli identified within each range.

Garner (1953, 1962, Ch. 3), among others, has drawn an analogy between the limited information per trial in category judgements (Figure 11) and the channel capacity that limits the transmission of information through an ideal communications system (Laming, 1968, Ch. 1; Weaver, 1949). This is inappropriate. Channel capacity is measured in bits/unit time, while the quantities in Figure 11 are bits per judgement. The two measures do not match dimensionally. Instead, the limit to information per trial in Figure 11 is a direct consequence of the relativity of judgement.

Calculating the variance of each identification within a set, *i* = 1, . . . *Ν* (Supplementary Material B, Equation B3),

(20) $$\begin{array}{l} \mathrm{Var}\left(\left(R_{i}-R_{\pi }\right)\;\;-\theta \left(R_{n-1}-R_{\pi }\right)\right)=\\ \theta^{2}\left[\sigma_{\beta }^{2}{\left(S_{i}-S_{n-1}\right)}^{2}+\sigma_{\varepsilon }^{2}\right] \end{array}$$

It includes a component θ^2^σ_β_^2^(*S_i_* − *S_n_*_−1_)^2^, increasing as the square of the difference between successive stimuli. When *S*_N_ is presented following *S*_1_ this component is (N-1)^2^θ^2^σ_β_^2^, increasing with the size of the set. Identification will begin to fail with end-of-set stimuli, when that variance, increasing in proportion to 1, 4, 9, 16, 25, . . . , becomes excessive. The limit appears to kick in at *N* = 4 or 5.

The dependence of accuracy on the size of the step between successive stimuli was demonstrated by Luce et al. (1982), who examined category judgements when the sequence of stimuli, of uniform frequency overall, was artificially constrained so that successive stimuli differed by small steps (−1, 0, 1), by slightly larger small steps (−2, −1, 0, 1,2), by large steps (⩾4) and compared these manipulations with a strictly random sequence of stimuli. Although the different stimuli were presented with equal frequency, small steps (−1, 0, 1) yielded much the best resolution and large steps (⩾4) the worst, worse even than strictly random. Values of *d*′ from these different conditions were modelled by Laming (1984, Table 3) using Equation 1 above.

The implications of relative judgement for a variety of experimental results were first pointed out by Laming (1984), working from Equation 1. That idea was applied to:

1. Stevens’ Power Law (cf. Laming, 1997, Ch. 11);
2. ME as implemented by S.S. Stevens and his colleagues, specifically the variance of numerical estimates (J. C. Stevens & Tulving, 1957; and data from S. S. Stevens, 1971, p. 441);
3. The autocorrelation of log numerical estimates (Baird et al., 1980);
4. The accuracy of category judgements, specifically the accuracy of resolution (*d*′ between adjacent stimuli) in the four experimental series studied by Luce et al. (1982);
5. The information per trial transmitted in category judgements.

However, the models in that paper were complicated by the involvement of Thurstonian scaling (Equation 14), which has here been abandoned. The present analyses are simpler.

Relativity of judgement has practical application to inspection tasks in general, including medical diagnosis (Laming, 2007), the marking of examination scripts (Laming, 1990, 2004a, Ch. 13; 2004b; Kelly et al., 2022), and even peer review (Laming, 1991). Because each judgement uses its predecessor as a point of reference, successive judgements have the propensity to wander. This can be especially important in medical screening, where a clinician makes a succession of judgements of very similar pathologies (Laming, 1995a, 1995b; Laming & Warren, 2000). In addition Laming (2004a) has collected a wide variety of examples of the relativity of judgement in contemporary society.

## Conclusion

This present experiment is the latest in a long sequence of studies of estimation/identification of a range of stimuli on a single continuum. Usually there are a small number of observers whose amalgamated results are presented as one. But the four observers in this experiment have each provided a sufficient volume of data to support individual analysis, and large differences can be seen between individual observers (Figures 2, 3, 5, 6, 9, and 10). Caution is needed in drawing conclusions that might apply to observers in general.

The observers in this experiment did not make absolute judgements; instead they referred each stimulus to its predecessor. Neither did they make absolute judgements of the difference/ratio between successive stimuli; although a greater stimulus difference led to a greater difference in successive responses, the extrapolation was variable. It is to be presumed that the observers were unable to accomplish either of these tasks and, to the extent that this experiment replicates the phenomena of similar studies, this presumption must apply to category judgement and ME in general.

The pattern of autocorrelation in Figure 3 has been used to account for the “bow” effect and the pattern of sequential effects and is therefore primary. It has been interpreted to signify that (a) there is no absolute judgement—each stimulus is judged relative to the stimulus and response on the preceding trial—and (b) there is likewise no absolute assessment of differences in stimulus magnitude—the adjustment from one trial to the next is variable.

The point of reference for each judgement therefore varies from trial to trial by the aggregation of independent increments and, by itself, that aggregation would ordinarily exceed all bounds. I have proposed a prior expectation to constrain the otherwise increasing variance, an expectation that is itself derived from the series of stimuli in the experiment. Successive points of reference nevertheless aggregate by small independent increments, and the aggregate distribution should be approximately normal. This provides an alternative explanation for the normal variation seen in ME and AI judgements and, indeed, in sensory judgement generally. Torgerson’s (1958) Case-V model is not needed and is better abandoned. Instead the analysis here has used only the ordinal metric of the stimulus set.

The ideas here contribute directly to an understanding of two other findings, external to the present experiment. Modelling in terms of the ordinal scale of the stimulus set accommodates range effects directly and the variance of judgements within that set provides an immediate explanation why observers are unable to identify more than five stimuli on a single continuum without error. Finally, there are practical applications to inspection tasks in general.

## Supplemental Material

sj-docx-1-qjp-10.1177_17470218231159393 – Supplemental material for Autocorrelation in category judgement Click here for additional data file. Supplemental material, sj-docx-1-qjp-10.1177_17470218231159393 for Autocorrelation in category judgement by Donald Laming in Quarterly Journal of Experimental Psychology

## Notes

1 Judging lengths of line as a way of explaining the magnitude estimation task was recommended by S. S. Stevens (1966, p. 531). There were 10 lines to be judged ranging from 12 to 150 mm in length.

2 So, while the first 10 stimuli duplicated Braida and Durlach’s procedure exactly, they were numbered in the inverse direction with respect to their original experiment.

3 This was in 1987/1989.

4 The original data are available on ResearchGate as Data.7z.

5 The principal results are presented below (Figures 3, etc.) for all four observers, but particular details of the analysis are illustrated with Observer 4 only.

6 To preempt a possible misunderstanding of Equation 1: Taking expectations in that equation,

$$E\left\{R_{n}\right\}\;=\;E\left\{R_{n-1}\right\}+\beta (S_{n}-S_{n-1})$$

which can be derived from

$$E\left\{R_{n}\right\}\;=\;c+\beta S_{n}$$

which, in turn, agrees very well with the mean response relationships in Figure 2. But *R_n_* and *R_n_*_−1_ are random variables, taking different values following different presentations of the stimuli to which they refer. Equation 1 says that when *S_n_* and *S_n-_*_1_ follow in succession, the values of *R_n_* and *R_n-_*_1_ are closely related and provides an adequate account of that relationship below.

7 The full set of parameter values for all four observers may be found in Tables 3a, b, c in Parameter_estimates.doc available on ResearchGate.

8 The reuse of the parameter θ, with identical value, is deliberate.

9 The analysis of sequential effects makes no appeal to the correction of “out of range” assessments (Equation 2) and *R_n_* and ***R***_*n*_ can be used interchangeably.

## References

[bibr1-17470218231159393] Bachem A. (1954). Time factors in relative and absolute pitch determination. Journal of the Acoustical Society of America, 26, 751–753.

[bibr2-17470218231159393] Baird J. C. Green D. M. Luce R. D. (1980). Variability and sequential effects in cross-modality matching of area and loudness. Journal of Experimental Psychology: Human Perception and Performance, 6, 277–289. 6445937 10.1037//0096-1523.6.2.277

[bibr3-17470218231159393] Berliner J. E. Durlach N. I. (1973). Intensity perception. IV. Resolution in roving-level discrimination. Journal of the Acoustical Society of America, 53, 1270–1287. 4712555 10.1121/1.1913465

[bibr4-17470218231159393] Braida L. D. Durlach N. I. (1972). Intensity perception. II. Resolution in one-interval paradigms. Journal of the Acoustical Society of America, 51, 483–502.

[bibr5-17470218231159393] Brown G. D. A. Neath I. Chater N. (2007). A temporal ratio model of memory. Psychological Review, 114(3), 539–576. 10.1037/0033-295X.114.3.539 17638496

[bibr6-17470218231159393] Brown S. D. Marley A. A. J. Dodds P. Heathcote A. (2009). Purely relative models cannot provide a general account of absolute identification. Psychonomic Bulletin & Review, 16(3), 583–593. 10.3758/PBR.16.3.583 19451389

[bibr7-17470218231159393] Brown S. D. Marley A. A. J. Lacouture Y. (2007). Is absolute identification always relative? Comment on Stewart, Brown, and Chater (2005). Psychological Review, 114(2), 528–532. 10.1037/0033-295X.114.2.528 17500642

[bibr8-17470218231159393] Durlach N. I. Braida L. D. (1969). Intensity perception. I. Preliminary theory of intensity resolution. Journal of the Acoustical Society of America, 46, 372–383. 5804107 10.1121/1.1911699

[bibr9-17470218231159393] Garner W. R. (1953). An informational analysis of absolute judgments of loudness. Journal of Experimental Psychology, 46, 373–380. 13109142 10.1037/h0063212

[bibr10-17470218231159393] Garner W. R. (1962). Uncertainty and structure as psychological concepts. Wiley.

[bibr11-17470218231159393] Garner W. R. Hake H. W. (1951). The amount of information in absolute judgments. Psychological Review, 58, 446–459. 14900305 10.1037/h0054482

[bibr12-17470218231159393] Gravetter F. Lockhead G. R. (1973). Criterial range as a frame of reference for stimulus judgment. Psychological Review, 80, 203–216. 4714675 10.1037/h0034281

[bibr13-17470218231159393] Green D. M. Luce R. D. Duncan J. E. (1977). Variability and sequential effects in magnitude production and estimation of auditory intensity. Perception & Psychophysics, 22, 450–456.

[bibr14-17470218231159393] Guest D. Adelman J. S. Kent C. (2016). Relative judgement is relatively difficult: Evidence against the role of relative judgement in absolute identification. Psychonomic Bulletin & Review, 23(3), 922–931. 10.3758/s13423-015-0940-2 26391032

[bibr15-17470218231159393] Guest D. Kent C. Adelman J. S. (2018). The relative importance of perceptual and memory sampling processes in determining the time course of absolute identification. Journal of Experimental Psychology: Learning, Memory & Cognition, 44(4), 615–630. 10.1037/xlm0000438 28967762

[bibr16-17470218231159393] Hake H. W. Garner W. R. (1951). The effect of presenting various numbers of discrete steps on scale reading accuracy. Journal of Experimental Psychology, 42, 358–366. 14888848 10.1037/h0055485

[bibr17-17470218231159393] Holland M. K. Lockhead G. R. (1968). Sequential effects in absolute judgment of loudness. Perception & Psychophysics, 3, 409–414.

[bibr18-17470218231159393] Hollingworth H. L. (1909). The inaccuracy of movement (Archives of Psychology, No. 13). The Science Press.

[bibr19-17470218231159393] Jesteadt W. Luce R. D. Green D. M. (1977). Sequential effects in judgments of loudness. Journal of Experimental Psychology: Human Perception and Performance, 3, 92–104. 845558 10.1037//0096-1523.3.1.92

[bibr20-17470218231159393] Kelly K. T. Richardson M. Isaacs T. (2022). Critiquing the rationales for using comparative judgement: A call for clarity. Assessment in Education: Principles, Policy & Practice, 29, 674–688. 10.1080/0969594X.2022.2147901

[bibr21-17470218231159393] Kent C. Lamberts L. (2005). An exemplar account of the bow and set-size effects in absolute identification. Journal of Experimental Psychology: Learning, Memory & Cognition, 31, 289–305. 15755246 10.1037/0278-7393.31.2.289

[bibr22-17470218231159393] Lacouture Y. (1997). Bow, range, and sequential effects in absolute identification: A response-time analysis. Psychological Research, 60, 121–133. 9411457 10.1007/BF00419760

[bibr23-17470218231159393] Laming D. (1968). Information theory of choice-reaction times. Academic Press.

[bibr24-17470218231159393] Laming D. (1984). The relativity of “absolute” judgements. The British Journal of Mathematical and Statistical Psychology, 37, 152–183.

[bibr25-17470218231159393] Laming D. (1990). The reliability of a certain university examination compared with the precision of absolute judgments. Quarterly Journal of Experimental Psychology, 42A, 239–254.

[bibr26-17470218231159393] Laming D. (1991). Why is the reliability of peer review so low? A comment on D.V. Cicchetti, H.O. Conn, and L.D. Eron, The reliability of peer review for manuscript and grant submissions: A cross-disciplinary investigation. Behavioral and Brain Sciences, 14, 154–156.

[bibr27-17470218231159393] Laming D. (1995a). The human element in medical screening. Journal of Medical Screening, 2, 52–55. 7497148 10.1177/096914139500200113

[bibr28-17470218231159393] Laming D. (1995b). Screening cervical smears. British Journal of Psychology, 86, 507–516. 8542199 10.1111/j.2044-8295.1995.tb02567.x

[bibr29-17470218231159393] Laming D. (1997). The measurement of sensation. Oxford University Press.

[bibr30-17470218231159393] Laming D. (1999). Prior expectations in cross-modality matching. Mathematical Social Sciences, 38, 343–359.

[bibr31-17470218231159393] Laming D. (2004a). Human judgment: The eye of the beholder. Thomson Learning.

[bibr32-17470218231159393] Laming D. (2004b). Marking university examinations: Some lessons from psychophysics. Psychology Learning and Teaching, 3(2), 9–16.

[bibr33-17470218231159393] Laming D. (2007). Ordinary people do not ignore base rates. A commentary on Barbey, A.K. and Sloman, S.A., Base-rate respect: From ecological rationality to dual processes. Behavioral and Brain Sciences, 30, 272–274. 10.1017/S0140525X07001653 17963533

[bibr34-17470218231159393] Laming D. (2010). Statistical information and uncertainty: A critique of applications in experimental psychology. Entropy, 12, 720–771. 10.3390/e12040720

[bibr35-17470218231159393] Laming D. Warren R. M. L. (2000). Improving the detection of cancer in the screening of mammograms. Journal of Medical Screening, 7, 24–30. 10807143 10.1136/jms.7.1.24

[bibr36-17470218231159393] Lockhead G. R. (2004). Absolute judgments are relative: A reinterpretation of some psychophysical ideas. Review of General Psychology, 8(4), 265–272.

[bibr37-17470218231159393] Lockhead G. R. Hinson J. (1986). Range and sequence effects in judgment. Perception & Psychophysics, 40, 53–61. 3748766 10.3758/bf03207594

[bibr38-17470218231159393] Luce R. D. Green D. M. (1974). The response-ratio hypothesis for magnitude estimation. Journal of Mathematical Psychology, 11, 1–14.

[bibr39-17470218231159393] Luce R. D. Green D. M. (1978). Two tests of a neural attention hypothesis for auditory psychophysics. Perception & Psychophysics, 23, 363–371. 683821 10.3758/bf03204138

[bibr40-17470218231159393] Luce R. D. Green D. M. Weber D. L. (1976). Attention bands in absolute identification. Perception & Psychophysics, 20, 49–54.

[bibr41-17470218231159393] Luce R. D. Nosofsky R. M. Green D. M. Smith A. F. (1982). The bow and sequential effects in absolute identification. Perception & Psychophysics, 32, 397–408. 7162940 10.3758/bf03202769

[bibr42-17470218231159393] Mori S. Ward L. M. (1995). Pure feedback effects in absolute identification. Perception & Psychophysics, 57(7), 1065–1079. 8532496 10.3758/bf03205465

[bibr43-17470218231159393] Nosofsky R. M. (1986). Attention, similarity, and the identification-categorisation relationship. Journal of Experimental Psychology: General, 115, 39–57. 2937873 10.1037//0096-3445.115.1.39

[bibr44-17470218231159393] Siegel W. (1972). Memory effects in the method of absolute judgment. Journal of Experimental Psychology, 94, 121–131.

[bibr45-17470218231159393] Stevens J. C. Tulving E. (1957). Estimations of loudness by a group of untrained observers. American Journal of Psychology, 70, 600–605. 13487830

[bibr46-17470218231159393] Stevens S. S. (1966). A metric for the social consensus. Science, 151, 530–541. 5323509 10.1126/science.151.3710.530

[bibr47-17470218231159393] Stevens S. S. (1971). Issues in psychophysical measurement. Psychological Review, 78, 426–450.

[bibr48-17470218231159393] Stewart N. Brown G. D. A. Chater N. (2005). Absolute identification by relative judgment. Psychological Review, 112, 881–911. 16262472 10.1037/0033-295X.112.4.881

[bibr49-17470218231159393] Tanner T. A. Rauk J. A. Atkinson R. C. (1970). Signal recognition as influenced by information feedback. Journal of Mathematical Psychology, 7, 259–274.

[bibr50-17470218231159393] Thurstone L. L. (1927a). A law of comparative judgment. Psychological Review, 34, 273–286.

[bibr51-17470218231159393] Thurstone L. L. (1927b). Psychophysical analysis. American Journal of Psychology, 38, 368–389. 3322058

[bibr52-17470218231159393] Torgerson W. S. (1958). Theory and methods of scaling. Wiley.

[bibr53-17470218231159393] Treisman M. (1985). The magical number seven and some other features of category scaling: Properties for a model of absolute judgment. Journal of Mathematical Psychology, 29, 175–230.

[bibr54-17470218231159393] Ward L. M. (1979). Stimulus information and sequential dependencies in magnitude estimation and cross-modality matching. Journal of Experimental Psychology: Human Perception and Performance, 5, 444–459. 528951 10.1037//0096-1523.5.3.444

[bibr55-17470218231159393] Ward L. M. Lockhead G. R. (1970). Sequential effects and memory in category judgments. Journal of Experimental Psychology, 84, 27–34.

[bibr56-17470218231159393] Ward L. M. Lockhead G. R. (1971). Response system processes in absolute judgment. Perception & Psychophysics, 9, 73–78.

[bibr57-17470218231159393] Weaver W. (1949). Recent contributions to the mathematical theory of communication. In Shannon C. E. Weaver W. (Eds.), The mathematical theory of communication (pp. 93–117). University of Illinois Press.

[bibr58-17470218231159393] Weber D. L. Green D. M. Luce R. D. (1977). Effects of practice and distribution of auditory signals on absolute identification. Perception & Psychophysics, 22, 223–231.

