# Supplementary material for: Autocorrelation in category judgement
Source: Q J Exp Psychol (Hove). 2023 Mar 24;76(12):2865–83. doi: 10.1177/17470218231159393 (PMC10655700; doi:10.1177/17470218231159393)
Supplement: sj-docx-1-qjp-10.1177_17470218231159393 – Supplemental material for Autocorrelation in category judgement [file sj-docx-1-qjp-10.1177_17470218231159393.docx]

Supplementary Material for:

Autocorrelation in category judgment

**Donald Laming**

University of Cambridge

Requests for reprints should be sent to Donald Laming at the University of Cambridge, Department of Psychology, Downing Street, Cambridge, England CB2 3EB.

E-mail: drjl@cus.cam.ac.uk

Supplementary Material A

autocorrelation

The basic response equation of relative judgment is

$R_{n} = R_{n-1}+B_{n}\text{(}S_{n}-S_{n-1}\text{)}+\varepsilon_{n}$, (A1)

The analyses in Figure 3 assemble individual trials from 30 different blocks, which may vary for a variety of reasons; ε*_n_* in Equation A1 represents that variance. The term
*B_n_*(*S_n_* – *S_n_*_-1_) is the adjustment needed for a stimulus different from its predecessor and, because *B_n_* is a random variable (there is neither any absolute judgment of stimulus differences), it adds an additional variance σ_β_^2^(*S_n_* – *S_n_*_-1_)^2^ to the response, proportional to the square of the difference, reducing the correlation coefficient for larger stimulus differences.

The correlations are modelled as follows:

$\begin{matrix} \text{Cov (}R_{n}, R_{n-1}\text{) }=\text{ }E\left\{\left[R_{n}-E\left\{R_{n}\right\}\right]\left[R_{n-1}-E\left\{R_{n-1}\right\}\right]\right\} \\ =E\left\{\left[R_{n-1}+B_{n}(S_{n}-S_{n-1})+\varepsilon_{n}-E\left\{R_{n-1}\right\}-E\left\{B_{n}\right\}(S_{n}-S_{n-1})\right]\left[R_{n-1}-E\left\{R_{n-1}\right\}\right]\right\} \\ =Var(R_{n-1}) \end{matrix}$ (A2)

because *B_n_* is independent of *R_n_*_-1_ and ε*_n_* has zero mean.

$\begin{matrix} \text{Var(}R_{n}\text{) }=\text{ }E\left\{\left[R_{n-1}+B_{n}(S_{n}-S_{n-1})+\varepsilon_{n}-E\left\{R_{n-1}\right\}-E\left\{B_{n}\right\}(S_{n}-S_{n-1})\right]^{2}\right\} \\ =\text{Var(}R_{n-1}\text{)}+\sigma_{\beta}^{2}(S_{n}-S_{n-1})^{2}+\sigma_{\varepsilon}^{2} \end{matrix}$. (A3)

Hence

$\begin{matrix} \text{Corr(}R_{n}, R_{n-1}\text{) }=\text{ Cov(}R_{n}, R_{n-1}\text{)/}\sqrt{\text{Var(}R_{n}\text{)Var(}R_{n-1}\text{)}} \\ =\sqrt{\text{Var(}R_{n-1}\text{)/Var(}R_{n}\text{)}} \\ =\left\{1+\left[\sigma_{\beta}^{2}(S_{n}-S_{n-1})^{2}+\sigma_{\varepsilon}^{2}\right]\text{/Var(}R_{n-1}\text{)}\right\}^{-1/2} \end{matrix}$, (A4)

In the case that a stimulus is repeated,

$\text{Corr(}R_{n}, R_{n-1}\text{) }=\text{ }\left\{1+\sigma_{\varepsilon}^{2}\text{/Var(}R_{n-1}\text{)}\right\}^{-1/2}$. (A5)

For a suitable choice of parameter values [*R_n_*_-1_, σ_β_^2^, σ_ε_^2^], Equation A4 fits the correlations for |*S_n_* – *S_n_*_-1_| ≤ 3 well (dashed curves in Figure 3), but for larger stimulus differences the skirts of that equation are all too high.

This problem occurs at extreme values of |*S_n_* – *S_n_*_-1_| and arises because initial assessments, *R_n_*, less than 1 or exceeding 10 are forced to ‘1’ or ‘10’ (see Fig. 4). This generates an excess in the observed distribution of ***R***_1_ relative to the prediction from Equation A1 and a deficit in ***R***_10_. The excess/deficit will be greatest when the difference between successive stimuli is large. Accordingly the under/overshoot is modelled with

$R_{n} = R_{n}-\alpha\text{(}S_{n}-S_{n-1}\text{)}$, (A6)

giving a positive correction when *S_n_* is less than *S_n_*_-1_, greatest when *S_n_* = *S*_1_ and a negative correction when *S_n_* is greater than *S_n_*_-1_, greatest when *S_n_* = *S*_10_.

The argument above is now modified as follows: First, *R_n_* in Equation A1 is replaced with *R_n_*–α(*S_n_* – *S_n_*_-1_). Second, *R_n_*_-1_, is equally subject to under/overshoot (Eq. 2). Averaging over the possible values of *S_n_*_-2_,

$E\left\{R_{n-1}\right\} = R_{n-1}-\alpha\text{(}S_{n-1}-\bar{S}\text{),}$ (A7)

where $\bar{S}$ is the average of the *S_n_*_-2_. According to Equation A6, the effect of that under/overshoot on *R_n_* depends on the location of *S_n_* in the stimulus set, specifically on $\text{(}S_{n}-\bar{S}\text{)}$. So *R_n_*_-1_ in Equation A2 must be replaced by $R_{n-1} +\alpha\text{(}S_{n}-\bar{S}\text{)}$ – ‘+’ because under/overshoot in *R_n_*_-1_ has the contrary effect on *R_n_*. Recalculating the covariance in Equation A2, for the recorded responses ***R****_n_*, ***R****_n_*_-1_,

$\text{Cov (}R_{n}, R_{n-1} +\alpha(S_{n}-\bar{S}=\text{ }E\left\{\left[R_{n}-E\left\{R_{n}\right\}-\alpha(S_{n}-S_{n-1})\right]\left[R_{n-1}-E\left\{R_{n-1}\right\}+\alpha(S_{n}-S_{n-1})\right]\right\}=E\left\{\left[R_{n}-E\left\{R_{n}\right\}\right]\left[R_{n-1}-E\left\{R_{n-1}\right\}\right]\right\}-\alpha^{2}(S_{n}-S_{n-1})^{2}=Var(R_{n-1})-\alpha^{2}(S_{n}-S_{n-1})^{2}$ (A8)

and

$\text{Corr(}R_{n}, R_{n-1}\text{) }=\text{ }\left[Var(R_{n-1})-\alpha^{2}(S_{n}-S_{n-1})^{2}\right]\text{/}\sqrt{\text{Var(}R_{n}\text{)Var(}R_{n-1}\text{)}}$. (A9)

Here ***R****_n_*,***R****_n_*_-1_ are the responses actually observed and *R_n_*, *R_n_*_-1_ the values prescribed by Equation 1. Equation A9 generates the curved characteristics in Figure 3, with the parameter values in Table 1.

In FB feedback ostensibly substitutes *S_n_*_-1_ for ***R****_n_*_-1_. Substituting *S _n_*_-1_ for ***R*** *_n_*_-1_ in Equation A8,

$\begin{matrix} \text{Cov (}R_{n}, S_{n-1}+\alpha\text{(}S_{n} -S_{n-1}\text{))} \\ \text{ }=\text{ }E\left\{\left[R_{n}-E\left\{R_{n}\right\}-\alpha(S_{n}-S_{n-1})\right]\left[S_{n-1}-E\left\{S_{n-1}\right\}+\alpha(S_{n} -S_{n-1})\right]\right\} \\ =-\alpha^{2}(S_{n}-S_{n-1})^{2}, \end{matrix}$ (A10)

because Cov(***R****_n_*, *S_n_*_-1_) = 0, giving

$\text{Corr(}R_{n}, S_{n-1}\text{) }=-\text{ }\left[\alpha^{2}(S_{n}-S_{n-1})^{2}\right]\text{/}\sqrt{\text{Var(}R_{n}\text{)Var(}S_{n-1}\text{)}}$; (A11)

Supplementary Material B

The ‘bow’ effect

Estimates of *d*' for the discrimination between adjacent stimuli (*S_i_* vs *S_i_*_+1_) show a depression in the middle of the stimulus set; this ‘bow’ results from an increase in response variance in the centre of the set (c.f. Fig. 5).

To derive an expression for response variances, first rewrite Equation 4 with a correction for overshoot at each end of the response scale,

$\left(R_{n}-R_{\pi}\right) = \theta\left[\left(R_{n-1}-R_{\pi}\right)+(B_{n}-\alpha)\text{(}S_{n}-S_{n-1}\text{)}+\varepsilon_{n}\right]$. (B1)

Taking expectations with respect to *S_n_*_-1_ = *S_j_*,

. (B2)

The right hand side of Equation B2 is independent of *S_n_*, so that the left hand side depends, at most, on *S_j_*. But the right hand side can be derived from Equation B1 with *n*-1 substituted for *n*. It is, at most, dependent on *S_n_*_-2_, and is otherwise independent of *S_j_*. It follows that the left hand side of Equation B2 is constant.

Calculating a variance from Equation B1,

$\text{Var}(\left(R_{n}-R_{\pi}\right) - \theta\left(R_{n-1}-R_{\pi}\right))=\theta^{2}\left[\sigma_{\beta}^{2}\text{(}S_{n}-S_{n-1}\text{)}^{2}+\sigma_{\varepsilon}^{2}\right],$ (B3)

because α(*S_n_–S_n_*_-1_) is fixed and does not contribute. But

$\begin{matrix} \text{Var}(\left(R_{n}-R_{\pi}\right) - \theta\left(R_{n-1}-R_{\pi}\right)) \\ =\text{Var}(\left(R_{n}-R_{\pi}\right))-2Cov(\left(R_{n}-R_{\pi}\right), \theta\left(R_{n-1}-R_{\pi}\right))+\theta^{2}\text{Var}(\left(R_{n-1}-R_{\pi}\right), \end{matrix}$ (B4)

and, adjusting the formula for covariance in Equation A8,

$\text{Cov (}\left(R_{n}-R_{\pi}\right), \theta\left(R_{n-1}-R_{\pi}\right)\text{) }=\theta\text{ }\left[Var(R_{n-1})-\text{½}\alpha^{2}(S_{n}-S_{n-1})^{2}\right]$. (B5)

Substituting in Equations B3 and B4,

$\begin{matrix} \text{Var}(R_{n}-R_{\pi})=2\theta\left[Var(R_{n-1}-R_{\pi})-\text{½}\alpha^{2}(S_{n}-S_{n-1})^{2}\right]- \\ \theta^{2}\text{Var}(R_{n-1}-R_{\pi})+\theta^{2}\left[\sigma_{\beta}^{2}\text{(}S_{n}-S_{n-1}\text{)}^{2}+\sigma_{\varepsilon}^{2}\right]. \end{matrix}$ (B6)

Consequent on the ordering of the stimuli, *S_n_* – any *S_n_* – is preceded equally often by all other stimuli, including itself. Averaging over the different ‘*n*–1’ trials within a set of *N* stimuli,

(B7)

where Var(*R_j_ – R*_π_) can be replaced by Var(***R****_j_ – R*_π_) since, according to Equation 2, ***R****_j_* and *R_j_* differ only by a fixed quantity. To this must be added, ordinarily, the variance of
E{***R****_n_*| *S_n_*_-1_ = *S_j_* } for *j* = 1, … 10, but the argument above (Eq. B2) shows that E{***R****_n_*| *S_j_*} is constant. Hence, substituting a particular stimulus for *S_n_*,

(B8)

where 8.25 is the average value of $\left(S_{j}-\bar{S}\right)()^{2}$. If overshoot be ignored (α = 0), then Equation B8 makes Var(*R_i_*) greatest at the extremes of the stimulus range (the contrary of the ‘bow’ effect). The concave curvature in Figure 5 therefore depends on $\left(\theta^{2}\sigma_{\beta}^{2}-\theta\alpha^{2}\right)<0$.

The data in Figure 5 are asymmetric, while Equation B8 is symmetric about $\bar{S}$. This asymmetry can be corrected, approximately, by writing *R*_π_ for $\bar{S}$.

$\begin{matrix} \text{Var}(R_{i}-R_{\pi})=N^{-1}\left(2\theta-\theta^{2}\right)\sum_{j} \text{Var}(R_{j}-R_{\pi})+ \\ \left(\theta^{2}\sigma_{\beta}^{2}-\theta\alpha^{2}\right)\left[\left(S_{i}-R_{\pi}\right)^{2}+8.25\right]+\theta^{2}\text{Var}(\varepsilon_{n}). \end{matrix}$ (B9)

Supplementary Material C

sequential effects

A clear understanding of sequential effects requires a multiple regression of ***R****_n_* on preceding stimuli and responses:

***R****_n_* = *c*+*a*_0_*S_n_* + *a*_1_*S_n_*_-1_+ *a*_2_*S_n_*_-2_+ *a*_3_*S_n_*_-3_…+*b*_1_***R****_n_*_-1_+ *b*_2_***R****_n_*_-2_+ *b*_3_***R****_n_*_-3_… + ε*_n_*. (C1)

Here *c* is a constant (with the effect of centring all variables to zero mean), ε*_n_* an error term, and the *a_k_* and *b_j_* regression coefficients estimated by minimisation of the sum of squares.

*Partial correlations with previous stimuli*

Rewriting Equation 6,

$\begin{matrix} R_{n}=\text{ }R_{\pi,n}+\theta\text{[}\left(R_{n\text{-1}}-\text{ }R_{\pi,n-1}\right)+B_{n}\text{(}S_{n}- S_{n\text{-1}}\text{)}+\varepsilon_{n}\text{] } \\ =\left(1-\theta\right)S_{n\text{-1}}+\theta\text{[}R_{n\text{-1}}+B_{n}\text{(}S_{n}- S_{n\text{-1}}\text{)}+\varepsilon_{n}\text{],} \end{matrix}$ (C2)

where $R_{\pi,n}-\theta R_{\pi,n-1}$has been substituted with $\left(1-\theta\right)S_{n\text{-1}}$ (Eq. 7). Previous responses *R_n-k_* can be eliminated from Equation C2 as follows: Substitute *n*–*k* for *n*, multiply by θ*^k^* and sum over *k*:

$\sum_{k=0}^{\infty} \theta^{k}R_{n-k} =\left(1-\theta\right)\sum_{k=1}^{\infty} \theta^{k-1}S_{n-k}+ \sum_{k=0}^{\infty} \theta^{k+1}\left(R_{n-k-1}+B_{n-k}\text{(}S_{n-k}-S_{n-k-1}\text{)}+\varepsilon_{n-k}\right)$, (C3)

or

$R_{n} =\left(1-\theta\right)\sum_{k=1}^{\infty} \theta^{k-1}S_{n-k}+ \sum_{k=0}^{\infty} \theta^{k+1}\left(B_{n-k}\text{(}S_{n-k}-S_{n-k-1}\text{)}+\varepsilon_{n-k}\right)$, (C4)

because the ***R****_n_*-*_k_* cancel for *k* ≥ 1.

The coefficients in Equation C1 are the covariances (***R****_n_*, *S_n_*-*_k_*) and (***R****_n_*, ***R****_n_*-*_k_*) divided respectively by Var(*S_n-k_*)[[1]](#footnote-1) and Var(***R****_n-k_*). Calculating those coefficients:

, (C5a)

where $\bar{S}$ is the stimulus mean (5.5) and $\bar{R}$ the mean response.

(C5b)

where β is the mean of *B_n_*.

(C5c)

Repeating the calculation for *i* ≥ 2, *a_i_* = θ*a_i_*_-1_.

*Response variance*

The coefficients in Equations C5b & c need to be multiplied by √[Var(*S_n-k_*)/Var(***R****_n_*)] to generate partial correlations to match the data in Figure 9. Var(*S_n-k_*) is known (8.25) and an expression for Var(***R****_n_*), free of previous responses, can be obtained by a similar argument to Equation C4. From Equation C2,

$Var\left(R_{n}\right) = \theta^{2}\left[Var\left(R_{n-1}\right)+\sigma_{\beta}^{2}\text{(}S_{n}-S_{n-1}\text{)}^{2}+Var\left(\varepsilon_{n}\right)\right]$, (C6)

because *S_n_* and *S_n-_*_1_ are given and contribute no variance. as before, substitute *n*–*k* for *n*, multiply by θ^2^*^k^* and sum over *k*:

$\sum_{k=0}^{\infty} \theta^{2k}Var\left(R_{n-k}\right) = \sum_{k=0}^{\infty} \theta^{2\left(k+1\right)}\left(Var\left(R_{n-k-1}\right)+\sigma_{\beta}^{2}\text{(}S_{n-k}-S_{n-k-1}\text{)}^{2}+Var\left(\varepsilon_{n-k}\right)\right)$, (C7)

or

(C8)

because the Var(***R****_n_*-*_k_*) cancel for *k* ≥ 1. If the expression for Var(***R****_n_*) be now averaged over the different sequences of stimuli, the cross-product term cancels and

(C9)

This gives a scaling factor in Figure 9 equal to

$\left(\frac{\left[\left(1-\theta^{2}\right)/\theta^{2}\right]Var\text{(}S\text{)}}{2\sigma_{\beta}^{2}Var\text{(}S\text{)}+Var\left(\varepsilon\right)}\right)^{1/2}$. (C10)

*Partial correlations with previous responses*

The model fitted to the partial correlations ***R****_n_* with previous responses, ***R****_n-k_*, *k* = 1, … 10, in Figure 10 is a simple negative exponential

*b_k_* = *b*_1_θ*^k^*^-1^. (C11)

1. Var(*S_n-k_*) = 8.25 and is the same for all *k*. [↑](#footnote-ref-1)
